# Pharmacological and Genetic Targeting of Inflammatory Chemokine Receptors CCR1, CCR2, and CCR5 in Atherosclerosis: A Systematic Review and Meta‐Analysis of Preclinical Studies

**DOI:** 10.1161/JAHA.125.041983

**Published:** 2026-02-20

**Authors:** Mohsen Shoaran, Elisabetta Caiazzo, Moustafa I. Morsy, Danila Gurgone, Neil MacRitchie, Dario Bruzzese, Armando Ialenti, Tomasz J. Guzik, Gerard J. Graham, Pasquale Maffia

**Affiliations:** ^1^ School of Infection & Immunity, College of Medical, Veterinary and Life Sciences University of Glasgow Glasgow United Kingdom; ^2^ Department of Pharmacy, School of Medicine and Surgery University of Naples Federico II Naples Italy; ^3^ Department of Public Health, School of Medicine and Surgery University of Naples Federico II Naples Italy; ^4^ BHF Centre for Research Excellence, Centre for Cardiovascular Sciences, Queen’s Medical Research Institute University of Edinburgh Edinburgh United Kingdom; ^5^ Department of Internal and Agricultural Medicine and Omicron Medical Genomics Laboratory Jagiellonian University College of Medicine Kraków Poland; ^6^ Africa‐Europe CoRE in Non‐Communicable Diseases & Multimorbidity African Research Universities Alliance (ARUA) & The Guild of European Research‐intensive Universities Glasgow United Kingdom

**Keywords:** atherosclerosis, CCR1, CCR2, CCR5, chemokine receptors, inflammation, meta‐analysis, Atherosclerosis, Vascular Disease

## Abstract

**Background:**

Inflammatory chemokine receptors encompassing CCR1, CCR2, CCR3, and CCR5 orchestrate the recruitment of leukocytes during inflammation. In atherosclerosis, there is ongoing controversy surrounding the precise role each inflammatory chemokine receptor plays in the disease process. We thus performed a systematic review and meta‐analysis to examine the effects of inflammatory chemokine receptor inhibition on experimental atherosclerotic burden.

**Methods:**

We performed a systematic literature search of PubMed/MEDLINE, Embase, and Web of Science for studies on the pharmacological and/or genetic manipulation of inflammatory chemokine receptors in murine models of atherosclerosis or hyperlipidemia. We extracted data on lesion size and morphological composition as primary outcomes, and plasma lipid profile and mouse body weight as secondary outcomes. We used a random effects model to calculate the pooled effect size across studies as the standardized mean difference (SMD) with 95% CIs.

**Results:**

A total of 38 studies of experimental atherosclerosis (CCR1: n=9, CCR2: n=24, CCR5: n=13) were included. Genetic or pharmacological inhibition of CCR2 and CCR5 significantly reduced lesion size (CCR2: SMD=−1.14 [95% CI, −1.47 to −0.81]; CCR5: SMD=−1.06 [95% CI, −1.64 to −0.48]), (CCR2: SMD=−0.72 [95% CI, −1.15 to −0.29]; CCR5: SMD=−1.92 [95% CI, −2.58 to −1.26]) and macrophage load (CCR2: SMD=−1.35 [95% CI, −1.97 to −0.74], CCR5: SMD=−1.29 [95% CI, −2.39 to −0.20]), (CCR2: SMD=−0.86 [95% CI, −1.43 to −0.29]; CCR5: SMD=−1.69 [95% CI, −2.69 to −0.69]), respectively. Pharmacological (but not genetic) targeting of CCR1 significantly reduced lesion size, with protection observed only in males when both approaches were considered.

**Conclusions:**

Our analysis suggests that inhibition of either CCR2 or CCR5 is protective in experimental atherosclerosis, while the effect of CCR1 intervention is less clear with potential beneficial effects in male populations.

**Nonstandard Abbreviations and Acronyms**
ApoE: apolipoprotein E
CCL: CC‐chemokine ligand
iCCR: inflammatory CC‐chemokine receptor
Ldlr: low‐density lipoprotein receptor
SMD: standardized mean difference

**Research Perspective**

**What Is New?**

- This meta‐analysis synthesizes preclinical evidence on targeting the inflammatory chemokine receptors CCR1, CCR2, and CCR5 in atherosclerosis.
- Genetic or pharmacological inhibition of either CCR2 or CCR5 is associated with a reduction in atherosclerotic burden (lesion size) in experimental models; inhibition of CCR2 or CCR5 also improves plaque stability, evidenced by reduced macrophage content.

**What Question Should Be Addressed Next?**

- Can simultaneous targeting of multiple inflammatory chemokine receptors, through genetic or pharmacological approaches, yield additive or synergistic effects in ameliorating atherosclerosis?

Cardiovascular diseases (CVD) constitute the leading cause of morbidity and mortality worldwide, with atherosclerosis being the most common underlying cause.^1^, ^2^, ^3^ While historically considered a passive disorder of vascular cholesterol accumulation, the multifaceted nature of atherosclerosis has since been delineated, with accompanying chronic inflammation acting as a key contributing factor to disease progression from early atherogenesis to plaque destabilization.^4^, ^5^, ^6^, ^7^, ^8^ A number of pivotal clinical trials have successfully shown that anti‐inflammatory intervention can reduce secondary cardiovascular events, particularly in subsets of patients presenting with residual inflammatory risk.^9^, ^10^, ^11^ As such, it has been proposed that targeting the inflammatory component of atherosclerosis may represent an attractive therapeutic strategy, potentially forming a refined treatment regimen incorporating conventional lipid‐lowering agents.^12^ Colchicine, one of the primary drugs whose effects were evaluated in these trials, has recently become the first anti‐inflammatory drug to gain Food and Drug Administration (FDA) approval for use in CVD.^13^

Trafficking and infiltration of leukocytes across the vascular wall, a key step in the initiation and progression of atherosclerotic lesions, is chiefly orchestrated by the interaction between endothelial and immune cell ligand‐receptor interactions including integrins, vascular adhesion molecules and chemokines.^14^, ^15^ CCR1, CCR2, CCR3, and CCR5 (collectively termed inflammatory CC‐chemokine receptors [iCCRs]), play an essential role in the regulation of myeloid cell recruitment during inflammation^16^, ^17^, ^18^ and are potential drivers of inflammatory diseases, such as rheumatoid arthritis^19^ and atherosclerosis.^14^ A growing body of literature supports an association between tissue or cell‐specific expression of iCCRs, as well as certain polymorphisms, and atherosclerosis in humans,^20^, ^21^, ^22^, ^23^, ^24^, ^25^, ^26^, ^27^, ^28^, ^29^ with a recent study linking genetic variation in CCR2 with myocardial infarction.^30^ Colchicine treatment significantly reduced transcoronary levels of the primary CCR2 ligand CC‐chemokine ligand (CCL2), which was found to be elevated in patients with acute coronary syndrome.^31^ However, although pilot studies have noted some beneficial effects of CCR5 inhibition on atherosclerotic parameters in patients with HIV at high cardiovascular risk,^32^, ^33^, ^34^ large randomized clinical trials using iCCR antagonists with defined cardiovascular end points are currently lacking. Consequently, there remains a strong reliance on preclinical studies in which receptors have been targeted through genetic or pharmacological inhibition.

iCCRs have been investigated extensively in various animal models of atherosclerosis, with a view to delineating their roles and their viability as therapeutic targets. CCR2 was the first iCCR to be linked with atherosclerosis, with early studies demonstrating that germline deletion of *Ccr2* ^35^ or *Ccl2* ^36^ in apolipoprotein E (Apoe)‐ and low‐density lipoprotein receptor (Ldlr)‐deficient mice, respectively, attenuated disease progression. Most follow‐up studies have generally observed similar trends following functional inhibition of the CCR2‐CCL2 axis in experimental atherosclerosis; a recent meta‐analysis that compiled 14 preclinical studies testing 11 different agents targeting CCR2 or CCL2 deduced an overall atheroprotective effect of pharmacological intervention.^37^ Still, there remains some variation in reported outcomes and underlying mechanisms that warrant further investigation, particularly considering the number of studies in which *Ccr2* has been genetically disrupted that have yet to be examined in detail. The precise contributions of CCR1 and CCR5 in experimental atherosclerosis are less clear, with various studies reporting protective, detrimental, or athero‐neutral effects of pharmacological inhibition or genetic knockdown of these receptors.^38^, ^39^, ^40^, ^41^, ^42^, ^43^ There are numerous factors that may contribute to these discrepancies, including the animal model or strain being used, intervention of choice, duration of intervention, and site or stage of lesion development that was examined. It is worth noting that iCCRs may occasionally be co‐expressed on immune cells and the situation is further confused by potential ligand promiscuity, although the extent to which these issues significantly impact iCCR biology is not clear.^16^

Given the scarcity of available data from the clinic, it is paramount that we develop a clearer understanding of the precise role of each iCCR from preclinical studies of atherosclerosis. Thus, herein we have performed a systematic review and meta‐analysis of preclinical studies reporting genetic and/or pharmacological inhibition of CCR1, CCR2, or CCR5 in murine experimental atherosclerosis, examining the effect of intervention on atherosclerotic lesion size or composition (ie, plaque macrophage, smooth muscle cell, and collagen content) as primary outcomes, as well as serum lipid profile (total cholesterol and triglyceride content) and body weight as secondary outcomes.

## METHODS

### Data Availability

The data supporting the findings of this study are available from the corresponding author upon reasonable request.

This meta‐analysis was performed according to the Preferred Reporting Items for Systematic Review and Meta‐Analyses (PRISMA) guidelines.^44^ The protocol of this study was registered in PROSPERO with registration number: CRD42024594213.

### Search Strategy

A systematic literature search was performed through PubMed/MEDLINE, Embase, and Web of Science databases from inception to September 2023 using both Medical Subject Headings (MeSH) and keywords limited to title and abstract related to studies assessing genetic and/or pharmacological manipulation of CCR1, CCR2, CCR3, and CCR5 receptors in experimental atherosclerosis. The search was restricted to articles published in English language. Details of the search strategy are available in Table S1. This work was done by 3 independent reviewers (M.S., M.I.M. and D.G.).

### Inclusion and Exclusion Criteria

#### Types of Studies

All studies were limited to original research. Any nonoriginal research studies such as reviews, editorials, commentaries, conference abstracts, case reports, and surveys were excluded. Human‐only and in vitro studies were also excluded.

#### Population

Mouse models of atherosclerosis and/or hyperlipidemia of any strain, age, and sex were included. Other disease model animals were excluded.

#### Types of Interventions

Any type of pharmacological or genetic manipulations targeting CCR1, CCR2, CCR3, or CCR5 receptors compared with a control were included. Studies that did not examine pharmacological or genetic targeting of CCR1, CCR2, CCR3, or CCR5 and studies not performed in mice were excluded.

### Outcomes

The primary outcomes were the quantitative analysis of atherosclerotic lesion area, neointimal area, or positive lipid‐staining area expressed as a percentage or a numerical value, as well as compositional measures of plaque vulnerability including the abundance of macrophages and smooth muscle cells, as determined by immunohistochemistry and lesion collagen content. Common histological analysis of the whole aorta or part of the aorta, such as the aortic root, aortic arch, aortic sinus, and thoracic/thoracoabdominal aorta, as well as brachiocephalic or carotid artery were acceptable. Where studies reported varying measurements of macrophages within the plaque, such as absolute and relative area, the latter was selected as the most robust measure. Secondary outcomes pertained to the effect of intervention on mouse body weight and lipid profiles including total cholesterol and triglyceride content. Studies without data on atherosclerotic lesion area or plaque composition were excluded.

### Study Selection

Three reviewers (M.S., M.I.M., and D.G.) independently screened titles and abstracts of the studies, after which full texts were reviewed. Disagreements on study selection were resolved by discussion and consensus.

### Data Extraction

Four reviewers (E.C., M.S., M.I.M., and D.G.) independently extracted data from included articles. Information recorded was tabulated and included: first author, year of publication, journal, experimental groups, control group(s), number of animals per group, absolute values, SD, or standard error (SE), vascular bed, species, exact genotype with genetic background, sex, weight, age, type of diet used, dose, timing of administration, frequency of administration, route of administration, vehicle, primary outcome(s) and secondary outcome(s). If data were not described numerically in text or tables, they were extracted from graphs or figures using PlotDigitizer (https://plotdigitizer.com/app). For missing data, authors were contacted. Where numbers of mice were reported as a range,^42^, ^45^, ^46^, ^47^, ^48^, ^49^, ^50^, ^51^, ^52^ the lower number was assumed for subsequent analysis. Disagreements or ambiguities were resolved by discussion and consensus.

### Study Quality Assessment

Four authors (E.C., M.S., M.I.M., and D.G.) assessed the risk of bias with the Systematic Review Centre for Laboratory Animal Experimentation (SYRCLE) tool.^53^ The tool is structured into a set of 10 standard domains: random sequence generation, baseline characteristics, allocation concealment, random housing, blinded interventions, random outcome assessment, blinding of outcome assessment, incomplete outcome data, selective reporting, and other sources of bias that were separately designated as either low, high, or unclear risk of bias. Publication bias was evaluated by visual inspection of funnel plots. Disagreements or ambiguities were resolved by discussion and consensus.

### Statistical Analyses

All statistical analyses were performed with R (4.4.0) software using (meta) package.^54^ Standardized mean differences (SMD) with 95% CIs as the effect size were calculated with Hedges method to account for small sample size. SMD was used because of the variation in outcome measurement units across the studies included in this analysis. In case SD was not reported, it was calculated using the reported SE and sample size. DerSimonian and Laird random effects models were used to account for the heterogeneity in the included studies, with the Hartung and Knapp (HK) correction to account for the small number of studies. The magnitude of heterogeneity between studies and subgroups was determined through visual inspection of the forest plots and then estimated using the Q‐test, I^2^, and τ^2^ statistic as moderate (I^2^ ≥50%) or high (I^2^ ≥75%). In addition, we also computed 95% prediction intervals to provide a range in which the true effect size of a future study would be expected to fall.^55^ To account for potential sources of heterogeneity, we performed subgroup analyses by vessel site, animal model, type of diet, duration of diet, and sex. A sensitivity analysis was also conducted by excluding studies deemed to be at high risk of bias. Finally, to assess the small study effect we produced funnel plots and assessed publication bias using Egger’s regression test. A *P* value < 0.05 was considered statistically significant.

## RESULTS

### Study Selection

**Figure 1:**
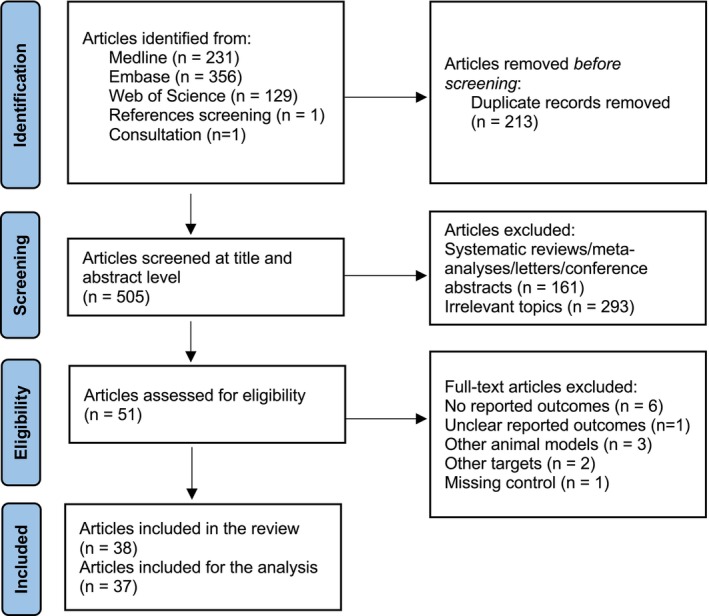
Preferred Reporting Items for Systematic Reviews and Meta‐Analyses flow diagram.

A flowchart detailing the study identification and selection process is illustrated in Figure 1. Our systematic search yielded a total of 716 articles, among which 213 were identified as duplicates. Two additional papers were identified through screening of references^51^ and manually searching journals that commonly publish studies on the subject of systematic review and meta‐analysis.^56^ Subsequently, upon screening titles and abstracts, 454 records were excluded for various reasons: 161 were either review articles, letters, editorials, meta‐analysesor conference abstracts, and 293 were deemed irrelevant to our topic. Following this initial screening, 51 studies were considered potentially eligible and their full texts were evaluated. Among these, 13 studies were subsequently excluded; 7 studies did not report outcomes of interest^57^, ^58^, ^59^, ^60^, ^61^, ^62^ or had unclear reported outcomes,^63^ 3 studies did not use experimental models of hyperlipidemia or atherosclerosis,^64^, ^65^, ^66^ 2 studies examined other (non‐iCCR) targets or used inappropriate interventions,^67^, ^68^ and 1 study failed to include a suitable control group.^69^ Ultimately, 38 articles were included in the systematic review, while the primary (meta‐) analysis was conducted on a total of 37 articles.

### Study Characteristics

The main characteristics of the included studies are described in Table S2. Most studies used a mouse model genetically predisposed to dyslipidemia and atherosclerosis, accompanied by feeding of a high‐fat (“Western”) diet or, less often, chow diet. The majority (n=29) of these studies used *Apoe* ^*−/−*^ mice, with 5 opting for *Ldlr* ^*−/−*^ ^41^, ^43^, ^70^, ^71^, ^72^ and 1 using ApoE3‐Leiden^73^ mice. Two further studies^40^, ^74^ investigated early atherosclerotic lesion formation in mice on a native C57BL/6 background fed an atherogenic diet.

Thirteen studies used models of accelerated or augmented atherosclerosis. Among these, 4 infused atheroprone mice with angiotensin II,^49^, ^75^, ^76^, ^77^ while 6 others reported outcomes following wire‐induced vascular injury^47^, ^50^, ^78^, ^79^, ^80^ or renal ischemia‐reperfusion injury.^71^ Cipriani et al^81^ treated mice with the boosted protease inhibitor ritonavir, which was associated with a proatherogenic shift in lipid profile and induced systemic inflammation. Lastly, 2 studies induced localized atherosclerosis via perivascular collar placement.^72^, ^82^

In studies where sex was specified, most outcomes of interest were reported in male mice. Eight studies did not clearly specify the sex of animals that were used.^35^, ^45^, ^47^, ^50^, ^52^, ^56^, ^77^, ^79^

Twenty‐one studies (55%) relied on genetic (global or hematopoietic) deletion of chemokine receptors.^35^, ^38^, ^39^, ^40^, ^41^, ^42^, ^46^, ^47^, ^50^, ^56^, ^71^, ^73^, ^74^, ^75^, ^78^, ^80^, ^83^, ^84^, ^85^, ^86^, ^87^ Among them, 5 reported targeting of *Ccr1*,^38^, ^40^, ^42^, ^50^, ^56^ thirteen intervened on *Ccr2*,^35^, ^42^, ^46^, ^71^, ^73^, ^74^, ^75^, ^78^, ^80^, ^83^, ^84^, ^86^, ^87^ and 7 on *Ccr5*.^38^, ^39^, ^41^, ^42^, ^47^, ^50^, ^85^

In addition to deleting iCCRs, 2 studies also reported coincidental knockout of the other inflammatory chemokine receptors *Cxcr3* ^87^ and *Cx3cr1*,^88^ while another study used mice deficient in the latter’s ligand, *Cx3cl1*.^86^ One study utilized siRNA silencing of *Ccr2* mRNA,^52^ while another study used lentiviral shRNA targeting of hematopoietic *Ccr2*.^76^

Fourteen studies relied on pharmacological blockade of CCRs with a duration of treatment ranging from 3 to 14 weeks. Among these, 7 studies targeted CCR2 using inhibitory compounds.^51^, ^77^, ^78^, ^82^, ^89^, ^90^, ^91^ With respect to CCR5, 2 studies opted for maraviroc, a selective CCR5 antagonist.^70^, ^81^ van Wanrooij et al^72^ reported the use of TAK‐779 in the context of CCR5 inhibition and it was classified accordingly, although it has also been shown to bind CCR2.^92^ A dual antagonist of CCR5/CCR1, N‐terminal methionylated Regulated upon Activation, Normal T‐cell Expressed and Secreted (Met‐RANTES), was used in 4 studies.^43^, ^47^, ^80^, ^88^ A further 4 studies^45^, ^48^, ^49^, ^76^ relied on gene transfection with a plasmid encoding an N‐terminal deletion mutant of MCP‐1 (monocyte chemoattractant protein‐1), 7‐ND, inhibiting the MCP‐1/CCR2 interaction.

### Risk of Bias and Publication Bias of Studies

**Figure 2:**
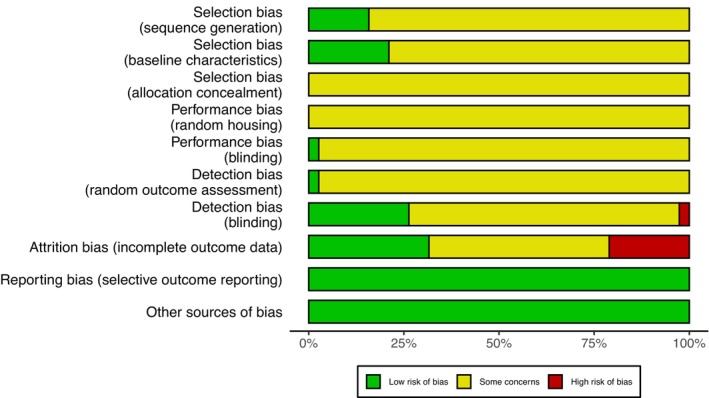
Risk of bias assessment according to Systematic Review Centre for Laboratory Animal Experimentation criteria.

The results of the Systematic Review Centre for Laboratory animal Experimentation risk of bias assessment for all studies included in our systematic review are outlined in Figure 2. Among the studies deemed eligible for inclusion, only 6 (16%) reported adequate generation of a randomized sequence assigned to each group, with a further 8 studies (21%) describing baseline characteristics including age, sex, and weight of animals. Allocation concealment and random housing domains were poorly reported across all studies and hence rated as unclear. Except for 1 study (3%), blinding of investigators from knowledge of interventions and random outcome assessment were similarly designated unclear in our assessment. Additionally, 1 study (3%) presented with a high risk of detection bias, where outcome assessment was not carried out under blinded conditions. We detected a high risk of attrition bias in a considerable segment of included studies (n=8, 21%) that failed to incorporate all experimental animals in downstream analyses with no explicit justification provided for excluding animals. While none of the studies prepublished or included a study protocol, all expected outcomes were found to be reported (comparing methods and results sections), resulting in a uniformly low risk of reporting bias. Furthermore, all studies were deemed to be low risk for other sources of bias. When additional interventions (eg, angiotensin II infusion) were present, they were incorporated into the experimental protocol and applied equally to both the experimental and control groups, ensuring the study was free from contamination because of pooled drugs.

For publication bias, visual inspection of funnel plots identified asymmetry in studies assessing the effect of receptor targeting on lesion size (Figure S1) and macrophage content (Figure S2) across CCR1, 2 and 5. Egger’s test showed a significant small‐study effect on lesion size in studies targeting CCR2 (β=−5.39 [95% CI, −7.07 to −3.70] *P*<0.001) and CCR5 (β=−5.09 [95% CI, −6.73 to −3.46] *P*<0.001), but not CCR1 (β=−3.10 [95% CI, −7.4 to 1.2] *P*=0.17) in the same analysis. Egger’s test was not appropriate for macrophage, smooth muscle cell, and collagen content attributable to the low number of studies available (k<10). By inspecting funnel plots, we investigated whether exclusion of studies deemed as outliers would affect the small study effect and the overall effect on lesion size. Excluding outlying studies targeting CCR2^35^, ^46^, ^51^, ^86^, ^93^ and CCR5^38^, ^43^, ^70^ attenuated the small‐study effect (CCR2, β=−4.24 [95% CI, −6.93 to −1.56] *P*=0.004; CCR5, β=−3.08 [95% CI, −5.67 to −0.5] *P*=0.02) with no major change on the pooled effect.

### Effect of Genetic and Pharmacological Targeting of Chemokine Receptors on Lesion Size, Plaque Composition, and Stability

**Figure 3:**
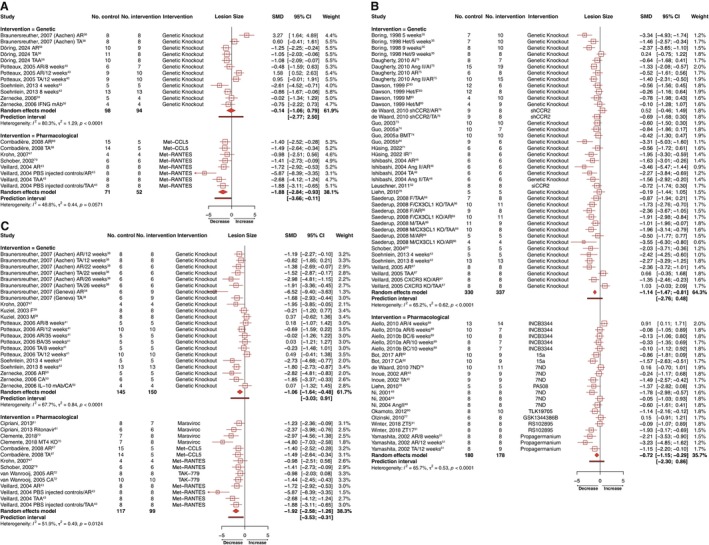
Forest plots showing the pooled estimate as a standardized mean difference (SMD) with corresponding 95% CIs and 95% prediction intervals for the effects of targeting (A) chemokine receptor 1, (B) chemokine receptor 2, and (C) chemokine receptor 5 on atherosclerotic lesion size. I^2^ and τ^2^ heterogeneity statistics are provided. (n) weeks indicates the duration on diet. AI indicates aortic intima; AngII, angiotensin II; AR, aortic root; BC, brachiocephalic artery; BMT, bone marrow transplantation; CA, carotid artery; CXCR3, C‐X‐C motif chemokine receptor 3; CX3CL1, chemokine C‐X3‐C motif ligand 1; F, female; Het, heterozygous; IFNG mAb, interferon gamma monoclonal antibody; IL‐10 mAb; interleukin‐10 monoclonal antibody; IR, ischemia reperfusion; M, male; MT4, metallothionein 4; TA, thoracic aorta; and TAA, thoracic/abdominal aorta.

Our meta‐analysis demonstrated that genetic targeting of the chemokine receptors CCR2 or CCR5 significantly reduced the mean size of lesions found across all vascular beds (CCR2: SMD=−1.14 [95% CI, −1.47 to −0.81], k=41, 337 experimental animals and 330 controls, Figure 3B; CCR5: SMD=−1.06 [95% CI, −1.64 to −0.48], k=22, 150 experimental animals and 145 controls, Figure 3C) but not CCR1 (CCR1: SMD=−0.14 [95% CI, −1.06 to 0.79], k=12, 94 experimental animals and 98 controls, Figure 3A). The 95% prediction intervals included the null for all targets, suggesting that the intervention’s effect may not be consistently detectable across experimental conditions comparable with those included in the present synthesis.

**Figure 4:**
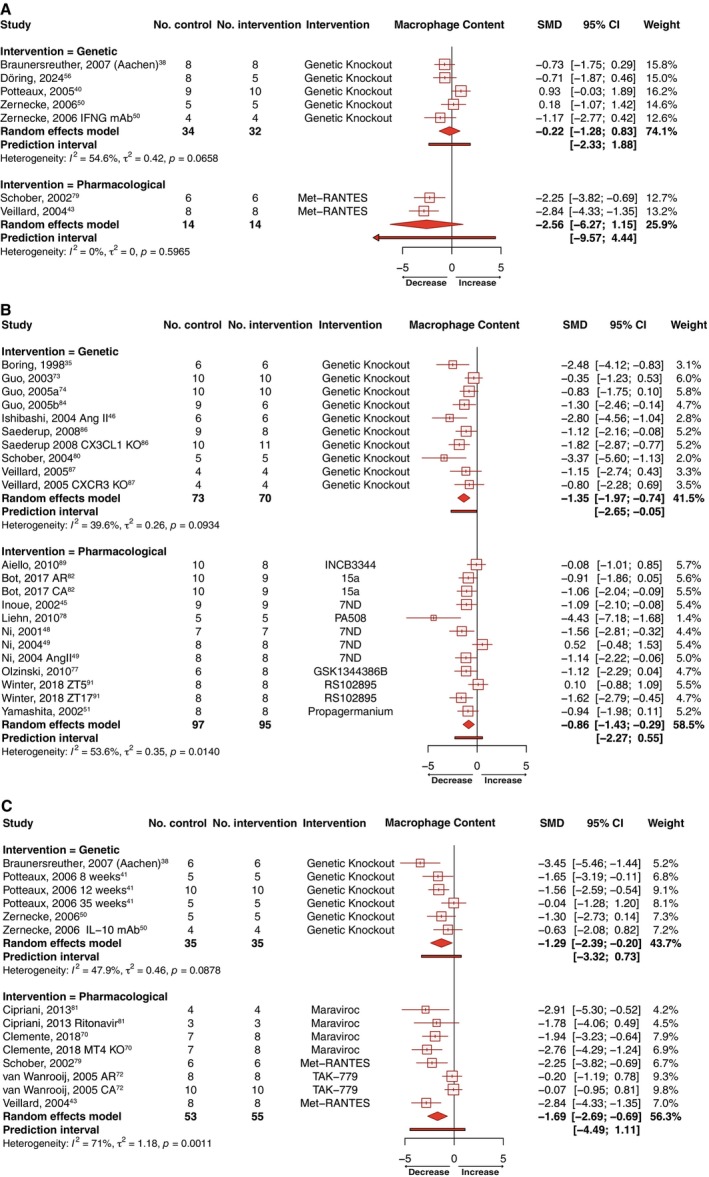
Forest plots showing the pooled estimate as a standardized mean difference (SMD) with corresponding 95% CIs and 95% prediction intervals for the effects of targeting (A) chemokine receptor 1, (B) chemokine receptor 2, and (C) chemokine receptor 5 on macrophage accumulation in atherosclerotic plaque. I^2^ and τ^2^ heterogeneity statistics are provided. (n) weeks indicates the duration on diet. AngII indicates angiotensin II; AR, aortic root; CA, carotid artery; CXCR3, C‐X‐C motif chemokine receptor 3; CX3CL1, chemokine C‐X3‐C motif ligand 1; IFNG mAb, interferon gamma monoclonal antibody; IL‐10 mAb; interleukin‐10 monoclonal antibody; and MT4, metallothionein 4.

However, pharmacological intervention yielded significant reductions in lesion size across the 3 receptors (CCR1: SMD=−1.88 [95% CI, −2.84 to −0.93], k=8, 52 experimental animals and 71 controls, Figure 3A; CCR2: SMD=−0.72 [95% CI, −1.15 to −0.29], k=21, 178 experimental animals and 180 controls, Figure 3B; CCR5: SMD=−1.92 [95% CI, −2.58 to −1.26], k=14, 99 experimental animals and 117 controls, Figure 3C). Prediction intervals excluded the null for CCR1 and CCR5 but crossed zero for CCR2, suggesting that the magnitude of the pharmacological effect may be less consistent for CCR2 across comparable experimental settings. Targeting of CCR2 or CCR5 alone were separately associated with significant reductions in lesional macrophage infiltration, both genetically (CCR2: SMD=−1.35 [95% CI, −1.97 to −0.74], k=10, 70 experimental animals and 73 controls, Figure 4B; CCR5: SMD=−1.29 [95% CI, −2.39 to −0.20], k=6, 35 experimental animals and 35 controls, Figure 4C) and pharmacologically (CCR2: SMD=−0.86 [95% CI, −1.43 to −0.29], k=12, 95 experimental animals and 97 controls, Figure 4B; CCR5: SMD=−1.69 [95% CI, −2.69 to −0.69], k=8, 55 experimental animals and 53 controls, Figure 4C). Only the genetic targeting of CCR2 yielded a prediction interval consistently below the null. Inhibition of CCR1 had no effect on macrophage content irrespective of designated intervention (Figure 4A). Suppression of CCR1, CCR2, or CCR5 was not associated with significant changes in either intraplaque smooth muscle cell (Figure S3) or collagen (Figure S4) content, although a trend toward increased collagen content and smooth muscle cell was apparent in the case of CCR2 inhibition (Figures S3B and S4B). Although not included in our meta‐analysis as standard error values were not reported, Quinones et al^85^ additionally described a reduction in lesion size associated with both global or hematopoietic deletion of CCR5 in advanced atherosclerotic *ApoE*‐null mice fed either chow or high‐fat diet, as well as reduced macrophage accumulation in *Ccr5* ^*−/−*^ animals fed normal laboratory diet.

Sensitivity analyses were conducted to ascertain whether and to what extent the inclusion of studies rated as high risk of bias (for 1 or more Systematic Review Centre for Laboratory Animal Experimentation domains) modified the pooled effect of receptor targeting on atherosclerotic lesion size (Figure S5). The exclusion of 8 such studies from our analysis had a low‐to‐moderate impact on effect size across CCR1 and CCR5 but did not alter our conclusions.

We further meta‐analyzed the association between genetic or pharmacological inhibition of iCCRs and defined secondary outcomes; manipulation of CCR1, CCR2, and CCR5 had negligible effects on mouse body weight (Figures S6A through S8A). Manipulation of CCR2 influenced lipid metabolism, with pharmacological blockade reducing total cholesterol levels and genetic targeting lowering triglyceride concentrations (Figure S7B and S7C). Conversely, genetic targeting of CCR1 showed increased total cholesterol levels (Figure S6B). Mouse lipid profiles were not affected by targeting CCR5 (Figure S8B and S8C).

### Subgroup Differences Based on Intervention, Sex, Vascular Bed, Model, Type of Diet, and Stage of Atherosclerosis

**Figure 5:**
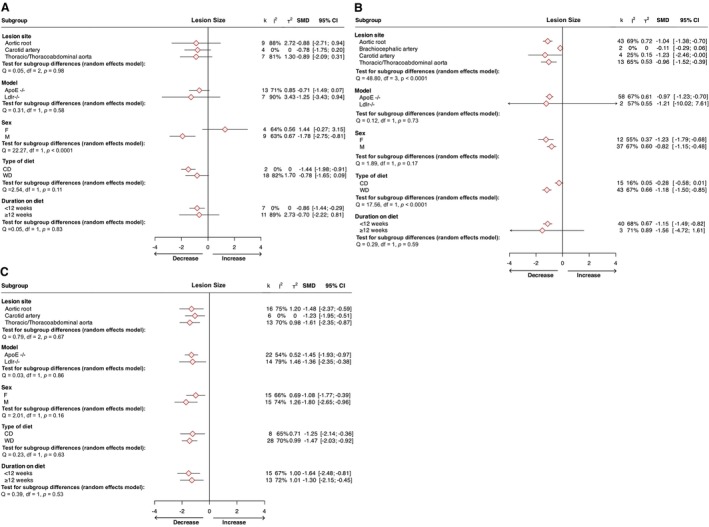
Subgroup analyses of targeting (A) chemokine receptor 1, (B) chemokine receptor 2, and (C) chemokine receptor 5 effect on lesion size by lesion site, mouse model, sex, and duration on diet. Standardized mean difference (SMD) and corresponding 95% CIs are provided with the number of studies (k) per group and heterogeneity between studies (I^2^, τ^2^). The differences between groups were assessed using Cochrane Q test. ApoE indicates apolipoprotein E; CD, chow diet; F, female; Ldlr, low‐density lipoprotein receptor; M, male; and WD, Western diet.

**Figure 6:**
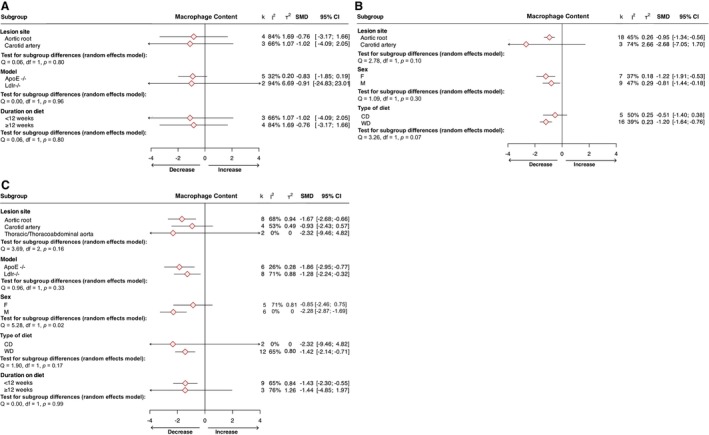
Subgroup analyses of targeting (A) chemokine receptor 1, (B) chemokine receptor 2, and (C) chemokine receptor 5 effect on macrophage accumulation in atherosclerotic plaque by lesion site, mouse model, sex, and duration on diet. Standardized mean difference (SMD) and corresponding 95% CIs are provided with the number of studies (k) per group and heterogeneity between studies (I^2^, τ^2^). The differences between groups were assessed using Cochrane Q test. ApoE indicates apolipoprotein E; CD, chow diet; F, female; Ldlr, low‐density lipoprotein receptor; M, male; and WD, Western diet.

In attempting to explain the moderate‐to‐high degree of heterogeneity (*I* ^2^) observed between studies across most primary outcomes and to uncover variable factors underlying these discrepancies, a series of subgroup analyses were performed. The outcome of targeting CCR2 (but not CCR1 or CCR5) on lesion area differed significantly between subgroups according to the site of lesion development assessed (*P*<0.0001), with the greatest reduction observed in the carotid artery (Figure 5B). Differences in the model of atherosclerosis used by studies were not associated with changes in the pooled effect of receptor inhibition on lesion size, with similar reductions in plaque burden apparent in *ApoE* ^*−/−*^ and *Ldlr* ^*−/−*^ mice subjected to CCR5 inhibition (in which the number of study arms was comparable across models; Figure 5C). Our analyses further revealed the outcome of CCR1 (but not CCR2 or CCR5) targeting on atherosclerotic burden was strikingly affected by sex (*P*<0.0001), with a significant reduction in lesion size exclusively found in male mice (SMD=−1.78 [95% CI, −2.75 to −0.81], k=9; Figure 5A). Separately, the decrease in macrophage content observed with CCR5 inhibition was also restricted to males (SMD=−2.28 [95% CI, −2.87 to −1.69], k=6; Figure 6C). No significant differences were discovered between subgroups on the effect of intervention on macrophage content according to lesion vascular bed or atherosclerosis model used across CCR1, CCR2, and CCR5 (Figure 6). Probing the influence of the type of diet fed to animals on the outcomes of meta‐analyzed studies, we demonstrated that the reductions in lesion size (Figure 5B) and macrophage content (Figure 6B) associated with inhibition of CCR2 were considerably larger in mice fed a Western diet compared with those fed normal laboratory chow, although the latter didn’t reach statistical significance. In contrast, differences in dietary composition were not associated with significant changes in the effect of CCR1 or CCR5 inhibition on lesion size (Figure 5A and 5C) or macrophage content (Figure 6A and 6C). Finally, we examined the influence of the stage of disease under assessment, dividing studies into those in which animals were fed a Western diet for less than or greater than 12 weeks as a proxy measure of earlier and more advanced atherosclerosis, respectively. While genetic or pharmacological inhibition of CCR5 significantly reduced lesion size across both stages of lesion development (Figure 5C), alleviations in lesion size on CCR1 (Figure 5A) or CCR2 (Figure 5B) inhibition were significant only in early stages of disease. Likewise, CCR5 inhibition‐associated reductions in lesion macrophage abundance were also significant only in earlier stages of disease (Figure 6C), although it is worth noting the relatively low number of study arms (k=3) reporting the effect of CCR5 targeting on macrophage content in late‐stage atherosclerosis.

## DISCUSSION

In this systematic review and meta‐analysis, we investigated the impact on atherosclerotic burden of targeting via genetic manipulation or receptor antagonism the inflammatory CC‐chemokine receptors 1, 2, and 5 in a total of 38 studies of experimental atherosclerosis, revealing apparent similarities as well as divergence in the role of iCCRs in atherosclerotic plaque formation. We found that inhibition of CCR2 and CCR5 were separately associated with a large reduction in lesion size, which was conserved across multiple vascular beds, occurring independently of intervention of choice (genetic versus pharmacological), accompanied by favorable changes in plaque composition including decreased macrophage abundance. Contrasting this, targeting of CCR1 was seen to alleviate lesion size (without changes in plaque composition), exclusively in the context of pharmacological intervention or, indeed, when male populations were considered in isolation. The absence of available studies reporting outcomes of interest following selective pharmacological or genetic inhibition of CCR3 in preclinical models of atherosclerosis precluded any analysis of the effect of its targeting.

iCCRs have been shown to play a fundamental role in the recruitment of myeloid cells, including monocytes and dendritic cells, to sites of inflammation^16^, ^18^ and, as such, represent potential therapeutic targets. In the setting of atherosclerosis, there persists a degree of controversy surrounding the contribution of each iCCR toward atherosclerosis development. This meta‐analysis provides evidence that ablation or antagonism of CCR2 and CCR5 exerts protective effects in experimental atherosclerosis. By contrast, the impact of CCR1 inhibition was less pronounced and may be context‐dependent or obscured by inter‐study variability. Although few, previous clinical trials using iCCR antagonists have demonstrated promise through positively modifying risk indicators of CVD. MLN1202, a humanized CCR2‐inhibiting monoclonal antibody, was used in a small‐scale phase II clinical trial of patients with CVD in which a significant reduction in circulating levels of high‐sensitivity C‐reactive protein, a biomarker of residual inflammation, was observed, as well as a reversible reduction in circulating monocytes compared with patients receiving placebo.^94^ Additionally, a phase IV clinical trial using the FDA‐approved CCR5 antagonist maraviroc demonstrated improvements in markers of CVD in patients with HIV at increased cardiovascular risk, with no reported change in systemic inflammation.^32^ Crucially, both drugs were well‐tolerated in enrolled patients. Consistent with what was reported in the latter trial,^32^ we noted a limited effect of iCCR antagonism on lipid profile (total cholesterol or triglyceride levels) in our analysis, indicating that the anti‐atherosclerotic effects of iCCR inhibition are likely mediated through immunomodulation. Our findings underline the potential utility of iCCR‐targeting drugs as part of a refined treatment regimen combating the residual inflammatory risk present in relevant patient subsets alongside conventional lipid‐lowering therapies. Still, while anti‐inflammatory strategies have shown considerable promise in treating atherosclerotic CVD, they are not without risk. For instance, the CANTOS (Canakinumab Anti‐inflammatory Thrombosis Outcome Study) trial showed that treatment with canakinumab, an IL‐1β inhibitor, was associated with an increased incidence of fatal infections and sepsis compared with placebo.^10^ Of relevance, inflammatory chemokine receptors play a central role in regulating leukocyte trafficking during immune responses, including in the context of infection. Although several clinical trials involving antagonists of these receptors have reported surprisingly good tolerability and favorable safety profiles,^32^, ^94^, ^95^ there remains a risk that their inhibition could impair host defense mechanisms, particularly during infection. This concern is heightened by the current lack of long‐term safety data for such agents in a cardiovascular setting. Precision medicine approaches characterizing the individual inflammatory profiles of patients could help identify those most likely to benefit from targeted chemokine receptor inhibition, while reducing potential risks.

iCCRs may occasionally be co‐expressed on the surface of immune cells^18^ and display elements of overlap with respect to ligand binding,^96^ potentially resulting in redundancy. If correct, this^16^ may limit the efficacy of targeting a single receptor and has been proposed as a key reason underlying the failure of previous clinical trials of receptor antagonists in a range of chronic inflammatory disorders.^97^ Given the potential off‐target effects of pharmacological agents, the fact that pharmacological inhibition was frequently seen to be superior to genetic deletion (particularly for CCR1 and CCR5) may argue for the benefits of multiple receptor targeting. Indeed, several pharmacological agents used in the included studies are known to exhibit multi‐receptor activity. Most notably, Met‐RANTES is reported to antagonize both CCR1 and CCR5, while TAK‐779 has strong binding affinity for CCR5 and CXCR3. Some studies included in our meta‐analysis also highlight compensatory interactions among chemokine receptors. For instance, van Wanrooij et al^72^ reported that treatment of Ldlr‐deficient mice with the CCR5/CXCR3 antagonist TAK‐779 led to a marked upregulation of *Ccr2* mRNA expression in the spleen, while Zernecke et al^50^ observed a reduction in CCR2^+^ cells in the neointima of CCR5‐deficient ApoE^−/−^ mice following arterial injury. These findings support the existence of inter‐receptor regulatory dynamics and reinforce the rationale for multi‐target therapeutic strategies in atherosclerosis. Nevertheless, other factors may also contribute to the observed differences between genetic and pharmacological approaches. In particular, compensatory mechanisms in knockout models could influence experimental outcomes. Genetic deletion often allows for functional adaptation during embryogenesis, whereas pharmacological inhibition is typically introduced acutely and may not permit such adaptation, potentially leading to a more pronounced immediate effect.

Preclinical studies investigating the combined role of CCR2 and CCR5 deficiency are lacking, partly because of the difficulty of generating compound‐receptor deficient mice attributable to genomic proximity of the genes encoding *iCCRs*.^16^ This precludes definitive deductions on the net contribution of these receptors to disease. However, there is ample evidence of specificity in the biological roles of CCR2 and CCR5 in atherosclerosis; for example, CCR5 expression has been shown to be elevated on *CCR2* ^*−/−*^ nonclassical monocytes, suggesting a degree of immune cell subset specificity.^62^ It may be that individual iCCRs play discrete roles in monocyte and macrophage recruitment at inflamed sites. For example, it is clear that CCR2 is the dominant receptor involved in monocyte extravasation to inflamed sites, and receptor expression analyses suggest that CCR1 and CCR5 may play roles of more importance in post extravasation cellular movement than in recruitment per se. In addition, a role for CCR5 in driving T cell migration to atherosclerotic lesions has recently been demonstrated in both mice^98^ and humans,^99^ with adoptive transfer of CD4 + CCR5+ effector cells exacerbating pathology in atherosclerotic animals.^98^ This additional capability of CCR5 to regulate lymphocyte homing in atherosclerosis could help explain the apparently larger effect size of overall CCR5 inhibition in comparison with CCR2. These differences in receptor function may further contribute to the possibility that simultaneous targeting of more than one iCCR could have a more pronounced atheroprotective effect than inhibiting a single receptor in isolation. In support of this proposition, pharmacological inhibition of CCR5 in mice genetically deficient in the primary CCR2 ligand, *Ccl2*, was reported to be additive in effect, resulting in an approximate 90% reduction of atherosclerosis when compared with *ApoE* ^*−/−*^ controls.^88^

In agreement with the meta‐analysis recently published by Živković et al,^37^ we report protective effects of pharmacological inhibition of CCR2 on atherosclerotic burden. Additionally, we show that pharmacological interventions are also effective in the context of CCR1 and CCR5 targeting. The overall congruity between the pooled effect trends of genetic and pharmacological interventions of CCR2 and CCR5 in reducing lesion size (and macrophage content) supports the efficacy of many of the pharmacological antagonists of these 2 receptors in successfully inhibiting iCCR function.

Our finding that CCR1 inhibition is protective exclusively in the context of pharmacological inhibition must be interpreted with caution because of the paucity of studies using selective CCR1 antagonists. Indeed, the sole CCR1‐targeting antagonist included in treatment arms, Met‐RANTES, has also been shown to bind CCR5 with a comparable degree of affinity.^43^, ^100^ Therefore, it cannot be deduced with any degree of certainty whether the reported outcomes of this treatment (significantly reduced atherosclerotic lesions) are mediated by its inhibition of CCR1, CCR5, or, indeed, both. Similarly, our observation that CCR1 targeting shows a sex‐specific effect, with interventions protective in males only, was also influenced by the effects of Met‐RANTES, with all such studies utilizing this compound (and where sex was reported), using male mice. Nevertheless, there also exists a clear discrepancy in the effect trend of *Ccr1* genetic deletion on lesion size between males and females, although the limited number of treatment arms necessitates a cautious interpretation of this observation. Evidence for sex differences in CCR1 function is scarce; however, some studies indicate that CCR1 expression may be elevated in female leukocytes, which could potentially modify receptor responsiveness to specific chemokines.^101^, ^102^ The reduction in lesion size associated with CCR1 inhibition was significant only at the earlier stage of disease. This finding is consistent with the work of Soehnlein et al.,^42^ who demonstrated stage‐specific effects of CCR1 deletion: macrophage numbers in the aorta were reduced early in disease (compared with *ApoE* *^⁻/⁻^* controls) but appeared to be increased at more advanced stages. Additionally, it has been proposed that the divergent effects of hematopoietic and global CCR1 deletion may also contribute to the discrepancies in reported outcomes.^15^ Further studies using *Ccr1‐*conditional knockout mice may help clarify if CCR1 has divergent cell‐specific roles in atherosclerosis.

To our knowledge, our study represents the first systematic review and meta‐analysis investigating the cumulative impact of targeting each of the iCCRs in experimental atherosclerosis and may offer insights into the design of future clinical trials in terms of target selection. However, there are also limitations to this study. First, our analysis revealed considerable heterogeneity of the effects of iCCR inhibition across most outcomes, which could potentially impact the derived effect estimates. This was reflected in the wide prediction intervals crossing zero, indicating that a null or even negative effect cannot be ruled out in future studies. While many of these differences are likely explained by the choice of intervention (genetic versus pharmacological), site of lesion formation, atherosclerotic model used, and stage of disease under investigation, there are additional complicating factors including: (1) pharmacological antagonists used in these studies that can differ considerably in their selectively, mode of action, dosage, and route of administration, and (2) strategy and associated efficiency of genetic suppression of *iCcr* function (eg, gene knockout versus siRNA silencing). Second, several of the individual subgroup analyses performed were relatively underpowered, further complicating interpretation of results.

In conclusion, preclinical evidence from our pooled analysis indicates the involvement of multiple chemokine receptors in atherosclerosis formation, primarily through the regulation of atherosclerotic lesion size and macrophage content within lesions. Inhibition of either CCR2 or CCR5 is protective in the context of atherosclerosis, while the associated effect of targeting CCR1 is less clear, with a potentially beneficial effect in early stages of disease that requires further investigation. Future preclinical studies should be strategically designed to include all iCCRs, enhancing our comprehension of their roles in atherosclerosis pathology.

## Sources of Funding

This work was supported by the British Heart Foundation grants (PG/19/84/34771, FS/19/56/34893A) awarded to P.M. P.M.’s laboratory is also supported by the British Heart Foundation grants (PG/21/10541, PG/21/10634, PG/23/11680 and PG/24/11946), by the Heart Research UK grant (SCOT24‐100004), the University of Glasgow and the International Science Partnership Fund, FRA 2020—Linea A University of Naples Federico II/Compagnia di San Paolo, the Italian Ministry of University and Research (MUR) PRIN 2022 (2022T45AXH) funded by the European Union—Next Generation EU, Mission 4, Component 1, CUP E53D23012760006, and the European Union ‐ Next Generation EU, Project CN00000041, Mission 4, Component 2, CUP B93D21010860004. TJG is supported by the European Research Council (ERC and InflammaTENSION, ERC‐CoG‐726 318); European Research Area—CVD (ERA‐CVD) (BrainGutImmune (ERA‐CVD/Gut‐brain/8/2021 and ImmmuneHyperCog, NCBiR Poland)), British Heart Foundation grants (FS/14/49/30838, FS/4yPhD/F/20/34127A and RE/18/5/34216). A.I. is supported by the Italian Ministry of University and Research (MIUR) PRIN 2020 (20203YAY9B). E.C. is supported by University of Naples Federico II (000005_CTB_PUG_CAIAZZO). M.I.M. is supported by the RS Macdonald Charitable Trust “Seedcorn Funding for Multidisciplinary Stroke Research” (Grant GA‐03503).

## Disclosures

None.

## Supporting information

Tables S1–S2
Figures S1–S8
