# Supplementary material for: Pharmacological and Genetic Targeting of Inflammatory Chemokine Receptors CCR1, CCR2, and CCR5 in Atherosclerosis: A Systematic Review and Meta‐Analysis of Preclinical Studies
Source: J Am Heart Assoc. 2026 Feb 20;15(5):e041983. doi: 10.1161/JAHA.125.041983 (PMC13055661; doi:10.1161/JAHA.125.041983)
Supplement: Supplementary file 1 — Tables S1–S2 Figures S1–S8 [file JAH3-15-e041983-s001.zip › jah370270-sup-0001-AppendixS1.pdf]

Supplemental Material

Table S1. Search strategy and keywords used to retrieve relevant studies.

**Disease**	"atherosclerosis" OR "atherosclerotic" OR "atherogenesis" OR "atherosclerosis-prone" OR "atheroprone" OR "atheroprogression"
**Population**	"LDLR" OR "mice lacking LDLR" OR "LDLR KNOCKOUT" OR "LDLR KO" OR "low-density lipoprotein receptor knockout" OR "LDLR deficient" OR "ApoE" "APOE KNOCKOUT" OR "APOE KO" OR "Apolipoprotein E knockout" OR "Apolipoprotein E deficient" OR "mice lacking apoE" OR "ApoE-LDLR double-deficient" OR "LDLR and ApoE double-deficient" OR "ApoE and LDLR double-deficient" OR "LDb" OR "Apolipoprotein B mRNA editing enzyme, catalytic polypeptide 1 and low-density lipoprotein receptor deficient" OR "Apoe*3-Leiden" OR "E3L" OR "Apolipoprotein E3-Leiden" OR "adeno-associated virus PCSK9" OR "adeno-associated virus protein convertase subtilisin/kexin type 9" OR "AAV-PCSK9" OR "AAV8-PCSK9" OR "Mus Musculus" OR "mice" OR "mouse" OR "murine" OR "rodents" OR "rodent"
**Intervention, exposure**	"inflammatory chemokine receptors" OR "iCCRs" OR "inflammatory CC chemokine receptors" OR "chemokine receptors"<br>"C-C motif chemokine receptor type 1" OR "C-C chemokine receptor type 1" OR "CC chemokine receptor type 1" OR "CCR1" OR "CC chemokine receptor 1" OR " CCR1/receptor" OR "CCR-1" OR "C-C motif chemokine receptor 1" OR "C-C chemokine receptor type 1" OR "MIP1aR" OR "CMKBR1" OR "SCYAR1" OR "CKR-1" OR "C-C CKR-1" OR "CC-CKR-1" OR "CD191" OR "Macrophage Inflammatory Protein 1-Alpha Receptor"<br>"C-C motif chemokine receptor type 2" OR "C-C chemokine receptor type 2" OR "CC chemokine receptor type 2" OR "CCR2" OR "CC chemokine receptor 2" OR "CCR2/receptor" OR "CCR-2" OR "CD192" OR "Monocyte Chemoattractant Protein 2 Receptor" OR "Monocyte Chemotactic Protein 2 Receptor" OR "MCP-1 Receptor" OR "CCR2A" OR "CCR2B" OR "CKR2A" OR "CKR2B"<br>"C-C motif chemokine receptor type 3" OR "C-C chemokine receptor type 3" OR "CC chemokine receptor type 3" OR "CCR3" OR "CC chemokine receptor 3" OR "CCR3/receptor" OR "CCR-3" OR "C-C motif chemokine receptor 3" OR "C-C chemokine receptor type 3" OR "CC-CKR-3" OR "CKR3" OR "CD193" OR "Eosinophil Eotaxin Receptor" OR "Eosinophil CC Chemokine Receptor 3" OR "B-Chemokine Receptor"<br>"C-C motif chemokine receptor type 5" OR "C-C chemokine receptor type 5" OR "CC chemokine receptor type 5" OR "CCR5" OR "CC chemokine receptor 5" OR "CCR5/receptor" OR "CCR-5" OR "C-C motif chemokine receptor 5" OR "C-C chemokine receptor type 5" OR "CC-CKR-5" OR "IDDM22" OR "CMKBR5" OR "CKR-5" OR "CD195" OR "CKR5" OR "HIV-1 Fusion Coreceptor"
**Outcome**	"plaque" OR "plaques" OR "lesion" OR "lesions" OR "atheroma" OR "atheromata" OR "macrophages" OR "macrophage" OR "necrotic core" OR "collagen" OR "vascular smooth muscle cells" OR "VSMCs" OR "smooth muscle cells" OR "SMCs" OR "fibrous cap" OR "intimal thickening" OR "intimal xanthoma" OR "fibrous cap atheroma" OR "calcification" OR "pathological intimal thickening" OR "IT" OR "IX" OR "PIT" OR "FCA"

Table S2. Main characteristics of the studies included in the systematic review and meta-analysis.

First author, year	Experimental groups	Control group (s)	Number of animals (Intervention)	Number of animals (control)	Lesion site	Genotype	Sex	Age at start of diet (wks)	Type of diet used	Duration of diet (wks)	Start of treatment (wks)	Dose	Duration of administration (wks)	Frequency of administration	Route of administration	Primary outcome(s)
Aiello, 2010[89]	INCB3344 (selective CCR2 antagonist)	Placebo (0.5% methyl cellulose in sterile saline)	14	13	Aortic root	C57BL/6, $Apoe^{-/-}$	M	NA	CD (Purina Prolab RMH 3000)	11	7	50 mg/kg	4	2x/daily	P.O.	Lesion size
			7	10						16	10		6			
			7	8						20			10			
			7	8	Brachiocephalic artery											
			8	10						26	20		6			
			8	10	Aortic root											MC
Boring, 1998[35]	$Ccr2^{-/-}$	$Ccr2^{+/+}$	6	6	Aortic root	C57BL/6-129/Sv, $Apoe^{-/-}$	NS	NA	WD (Harlan Tekland TD- no. 88137, 21% fat, 0.15% Cl)	5	NA	NA	NA	NA	NA	MC
	$Ccr2^{+/-}$		10	7												Lesion size
	$Ccr2^{-/-}$		10	7												
	$Ccr2^{+/-}$		8	8						9						
	$Ccr2^{-/-}$		10	8												
Bot, 2017[82]	15a (CCR2 antagonist)	Vehicle	9	10	Carotid artery	NA, $Apoe^{-/-}$	M	10-12	WD (SDS, 15% cocoabutter, 0.25% cholesterol)	6	12-14	5 mg/kg	4	Daily	I.P.	Lesion size
			9	10	Aortic root											
			9	10	Carotid artery											MC
			9	10												
			9	10	Aortic root											
			9	10												
Braunersreuther, 2007[38]	$Ccr5^{-/-}$	$Ccr5^{+/+}$	8	8	Aortic root	C57BL/6J, $Apoe^{-/-}$	F	9-12 for WD	WD (Altromin, 21% fat; 0.15% Cholesterol)	12	NA	NA	NA	NA	NA	Lesion size
			8	8	T/TAA											
			6	6	Aortic root					22						
			6	6	T/TAA											
			6	6	Aortic root				CD	26						
			6	6	T/TAA											
			8	8	Aortic root				WD (Altromin, 21% fat; 0.15% Cholesterol)	10 to 12						MC
			8	8												SMC content

First author, year	Experimental groups	Control group (s)	Number of animals (Intervention)	Number of animals (control)	Lesion site	Genotype	Sex	Age at start of diet (wks)	Type of diet used	Duration of diet (wks)	Start of treatment (wks)	Dose	Duration of administration (wks)	Frequency of administration	Route of administration	Primary outcome(s)
			9	6					WD (Research Diets D-12108, 20.1% fat, 1.25% cholesterol)	10						Lesion size
			9	6	T/TAA											
	Ccr1$^{-/-}$	*Ccr1*$^{+/+}$	8	8	Aortic root				WD (Altromin, 21% fat; 0.15% cholesterol)	12						
			8	8	T/TAA											
			8	8	Aortic root											MC
			8	8												SMC content
Cipriani, 2013[81]	Maraviroc (CCR5 antagonist)-+ Ritonavir (HIV protease inhibitor) -	Ritovanir -	6	6	T/TAA	C57BL/6J, *Apoe*$^{-/-}$	M	NA	CD	21	8	50 mg/kg	13	Daily	P.O.	Lesion size
			5	6												MC
			3	3						52	39					
			7	8												Lesion size
Clemente, 2018[70]	Maraviroc -(CCR5 antagonist)	Vehicle (5% dimethyl sulfoxide and 0.5% HCl 0.1 N in distilled water)	8	7	Aortic root	C57BL/6, *Ldlr*$^{-/-}$	M	12	WD (Ssniff, E15721-34)	8	12	25 µg/g	8	Every 12 h for 1st week, every 24 hr for further 7 weeks	P.O.	MC
			8	7		C57BL/6, *Mt4*$^{-/-}$, *Ldlr*$^{-/-}$										
			8	7	T/TAA	C57BL/6, *Ldlr*$^{-/-}$										Lesion size
			8	7		C57BL/6, *Mt4*$^{-/-}$, *Ldlr*$^{-/-}$										
Combadière, 2008[88]	Met-CCL5-CCR5 antagonist	vehicle	5	15	Aortic root	C57BL/6, *Apoe*$^{-/-}$, *Ccl2*$^{-/-}$, *Cx3cr1*$^{-/-}$	M	NA	CD	26	14	100 µg /w	12	2x/week	I.P.	Lesion size
			5	14	Thoracic aorta											
Daugherty, 2010[75]	*Ccr2*$^{-/-}$ (Saline)	*Ccr2*$^{+/+}$ (Saline)	7	8	T/TAA	C57BL/6, *Apoe*$^{-/-}$	M	NA	WD (0.15 % cholesterol and 21 % (w/w) milk fat diet TD 88137;	5	NA	NA	NA	NA	NA	Lesion size
	Ccr2$^{-/-}$ (Ang II)	*Ccr2*$^{+/+}$ (Ang)	19	15												
	Ccr2$^{-/-}$ (Saline)	*Ccr2*$^{+/+}$ (Saline)	8	6	Aortic root											
	Ccr2$^{-/-}$ (Ang II)	*Ccr2*$^{+/+}$ (Ang)	15	10												

First author, year	Experimental groups	Control group (s)	Number of animals (Intervention)	Number of animals (control)	Lesion site	Genotype	Sex	Age at start of diet (wks)	Type of diet used	Duration of diet (wks)	Start of treatment (wks)	Dose	Duration of administration (wks)	Frequency of administration	Route of administration	Primary outcome(s)
									Harlan Teklad)							
Dawson, 1999[83]	*Ccr2-/-*	*Ccr2+/+*	6	12	Aortic root	C57BL/6, *Apoe*$^{-/-}$	F	NA	CD (Purina Picolab 5058)	16	NA	NA	NA	NA	NA	Lesion size
	Ccr2+/-		8	12												
	Ccr2-/-		10	4			M									
	Ccr2+/-		9	4												
de Waard, 2010[76]	7ND	control	11	10	Aortic root	NA, *Apoe*$^{-/-}$		8 to 10	WD (SDS)	8 to 9	12-14	NA	4-5	once	In-vivo electroporation of femoral muscles	Lesion size
			11	10												Collagen content
	Ccr2$^{-/-}$ BM reconstitution with siRNA-mediated gene silenced BM	control	8	9	T/TAA			17			16-20				Lentiviral infection of BM cells I.V.	Lesion size
			8	9	Aortic root											
Döring, 2024[56]	*Ccr1*$^{-/-}$	*Ccr1*$^{+/+}$	8	10	Thoracic aorta	C57BL/6, *Apoe*$^{-/-}$	NA	8-10	WD (21% fat and 0.15–0.2% cholesterol (Altromin 132010, Sniff TD88137)	12	NA	NA	NA	NA	NA	Lesion size
			8	11	Aortic arch											
			9	10	Aortic root											
			5	8												MC
			5	7												SMC content
			5	7												Collagen content
Guo, 2003[73]	*Ccr2*$^{-/-}$ BM reconstitution	*Ccr2*$^{+/+}$ BM reconstitution	10	10	Aortic root	ApoE3-Leiden	F	14-16	WD (15% fat, 1% cholesterol, 0.5% cholate)	8	6-8	1 x 10^7 BM cells	16	once	I.V.	Lesion size
			10	10												MC
Guo, 2005 (a)[74]	*Ccr2*$^{-/-}$ BM reconstitution	*Ccr2*$^{+/+}$ BM reconstitution	10	10	Aortic root	NA, *Apoe*$^{-/-}$	F	NA	CD (RM3; Special Diet Services)	25	16	1 x 10^7 BM cells	9	once	I.V.	Lesion size
	Ccr2$^{-/-}$	*Ccr2*$^{+/+}$	7	10						24	NA	NA	NA	NA	NA	

First author, year	Experimental groups	Control group (s)	Number of animals (Intervention)	Number of animals (control)	Lesion site	Genotype	Sex	Age at start of diet (wks)	Type of diet used	Duration of diet (wks)	Start of treatment (wks)	Dose	Duration of administration (wks)	Frequency of administration	Route of administration	Primary outcome(s)
	Ccr2$^{-/-}$ BM reconstitution	*Ccr2*$^{+/+}$ BM reconstitution	10	10						25	16	1 x 10^7 BM cells	9	once	I.V.	Collagen content
			10	10												MC
Guo, 2005 (b)[84]	*Ccr2*$^{-/-}$ BM reconstitution	*Ccr2*$^{+/+}$ BM reconstitution	6	9	Aortic root	C57BL/6, *Ccr2*$^{-/-}$	**F**	6-8	WD (Hope Farms, 15% fat, 1% cholesterol, 0.5% cholate)	10	6-8	1 x 10^7 BM cells	18	once	I.V.	Lesion size
			6	9												MC
Hüsing, 2022[71]	*Ccr2*$^{-/-}$ BM reconstitution	*Ccr2*$^{+/+}$ BM reconstitution	6	6	Aortic root	C57BL/6, *Ldlr*$^{-/-}$	M	NA	WD (Harlan Teklad TD-88137)	10	NA	NA	NA	NA	NA	Lesion size
	Ccr2$^{-/-}$ BM reconstitution following IR	*Ccr2*$^{+/+}$ BM reconstitution following IR	6	8												
Inoue, 2002[45]	pcDNA3-7ND (plasmid encoding N terminal deletion mutant of MCP-1)	Control (empty plasmid)	9	9	Aortic root	C57BL/6J, *Apoe*$^{-/-}$	M	NA	CD	28	20	100 µg	8	Biweekly intervals	I.M.	Lesion size
			9	9	Aortic arch											MC
			9	9												Smooth Muscle Cells content
			9	9												Collagen content
Ishibashi, 2004[46]	*Ccr2*$^{-/-}$ BM reconstitution	*Ccr2*$^{+/+}$ BM reconstitution	6	6	T/TAA	C57BL/6J-129SvJae	M	NA	NA	NA	8	1 x 10^7 BM cells	10	once	I.V.	Lesion size
					Aortic root											MC
Krohn, 2007[47]	*Ccr5*$^{-/-}$	*Ccr5*$^{+/+}$	4	4	Carotid artery	NA, *Apoe*$^{-/-}$	NS	NA	WD (SDS, 15% cacao butter, 0.25% cholesterol	5	NA	NA	NA	NA	NA	Lesion size
	Met-RANTES (CCR5 antagonist)	*Ccr5*$^{+/+}$	4	4							NA	40 µg	4	3 times/week	NA	

First author, year	Experimental groups	Control group (s)	Number of animals (Intervention)	Number of animals (control)	Lesion site	Genotype	Sex	Age at start of diet (wks)	Type of diet used	Duration of diet (wks)	Start of treatment (wks)	Dose	Duration of administration (wks)	Frequency of administration	Route of administration	Primary outcome(s)
Kuziel, 2003[39]	*Ccr5*$^{-/-}$	*Ccr5*$^{+/+}$	8	8	Aortic root	C57BL/6, *Apoe*$^{-/-}$	F	NA	CD (Purina Picolab 5058)	16	NA	NA	NA	NA	NA	Lesion size
			8	8			M									
Leuschner, 2011[52]	siRNA nanoparticles targeting *Ccr2* (siCCR2)	control siRNA treatment (siCON)	8	8	Aortic root	B6.129P2-*Apoe*^{tm1Unc}	NS	8	WD (Harlan Tekland, 21.2% fat/weight, 0.2% cholesterol)	16	21	0.5 mg/kg	3	2x/week	I.V.	Lesion size
Liehn, 2010[78]	PA508 (CCR2 antagonist)	Vehicle	5	5	Aortic root	C57BL/6J, *Apoe*$^{-/-}$	F	NS	WD	NS	8	10 µg	3	Daily	I.P.	Lesion size
																MC
																SMC content
																Lesion size
Ni, 2001[48]	7ND (plasmid encoding terminal deletion mutant of monocyte chemoattractant protein-1) encapsulated in 7HVJ liposome	Control (PBS)	8	8	Aortic root	C57BL6/J, *Apoe*$^{-/-}$	F	7-8	WD (Oriental Yeast, 20% fat, 0.15% cholesterol)	6	7-8	5 µg	3-6	2x (weeks 0 and 3)	I.M.	Lesion size
			7	7												MC
			7	7												Collagen content
			7	7												SMC content
Ni, 2004[49]	pcDNA3 –7ND plasmid DNA	Control (pCDNA 3 plasmid DNA) + saline	8	8	Aortic root	C57BL6/J, *Apoe*$^{-/-}$	M	NA	CD (Oriental Yeast)	34	30	100 µg	4	1× biweekly intervals	I.M. of plasmid DNA (pcDNA 3-7ND) with	Lesion size
			8	8												MC
			8	8												SMC content
			8	8												Collagen content

First author, year	Experimental groups	Control group(s)	Number of animals (Intervention)	Number of animals (control)	Lesion site	Genotype	Sex	Age at start of diet (wks)	Type of diet used	Duration of diet (wks)	Start of treatment (wks)	Dose	Duration of administration (wks)	Frequency of administration	Route of administration	Primary outcome(s)
	7ND + Ang II	Control (pCDNA 3 plasmid DNA) + Ang II	8	8											electroporation	Lesion size
			8	8												MC
			8	8												SMC content
			8	8												Collagen content
Okamoto, 2012[90]	TLK19705 (CCR2 antagonist)	Control (drug-free diet)	10	8	Aortic root	C57BL/6, *Apoe*$^{-/-}$	M	4	WD (Oriental Yeast, 15% high-fat diet,1.25 % cholesterol)	8	4	10 mg/kg	8	Daily	Oral (admixed with diet)	Lesion size
Olzinski, 2010[77]	GSK1344386B(CCR2 antagonist)	Vehicle (drug-free diet)	8	6	Aortic root	*hu*CCR2ki/ *Apoe*$^{-/-}$	NS	22-24	WD (Harlan Tekland TD-88137,42 % of calories from fat and containing 21% anhydrous milkfat, 34% sucrose and 0.2% cholesterol)	5	22-24	10 mg/kg	5	Daily	Oral (admixed with diet)	Lesion size
			8	6												MC
Potteaux, 2005[40]	*Ccr1*$^{-/-}$ BM reconstitution	*Ccr1*$^{+/+}$ BM reconstitution	7	6	Aortic root	C57BL/6J, *Ldlr*$^{-/-}$	M	32	WD (15% fat, 1.25% cholesterol, and 0% cholate)	8	28	NA	12	once	I.V.	Lesion size
			10	9	T/TAA		F									
			10	9	Aortic root					12			16			
			10	9												MC
			10	9												SMC content
			10	9												Collagen content
Potteaux, 2006[41]	*Ccr5*$^{-/-}$ BM	*Ccr5*$^{+/+}$ BM	10	9	Aortic root	C57BL/6, *Ldlr*$^{-/-}$	M	19	WD (15% fat, 1.25%	12	15	2.5 x 10^6 BM cells	16	once	I.V.	Lesion size
			10	9			F			12			16			

First author, year	Experimental groups	Control group (s)	Number of animals (Intervention)	Number of animals (control)	Lesion site	Genotype	Sex	Age at start of diet (wks)	Type of diet used	Duration of diet (wks)	Start of treatment (wks)	Dose	Duration of administration (wks)	Frequency of administration	Route of administration	Primary outcome(s)
	reconstitution	reconstitution	10	9	Brachiocephalic artery				cholesterol, 0% cholate)	35			39			
			10	9	Aortic root		M			8			12			
			10	10			F			12			16			
			5	5						35			39			
			5	5			M			8			12			MC
			10	10			F			12			16			
			5	5						35			39			
			10	10						12			16			Collagen content
			5	5						35			39			
			10	10						12			16			SMC content
			5	5						35			39			
Quinones, 2007[85]	*Ccr5*$^{-/-}$	*Ccr5*$^{+/+}$	10	9	Aortic root	C57BL/6J, *Apoe*$^{-/-}$	M/F	NA	CD	26	NA	NA	NA	NA	NA	Lesion size
			15	27					CD	36-45						
			14	11					CD	65-80						
			16	12					WD (21% fat from lard and supplemented with 0.15% (w/w) cholesterol)	NA						
	Ccr5$^{-/-}$ BM reconstitution	*Ccr5*$^{+/+}$ BM reconstitution	15	18				NA	CD	36	6	NA	30	once	I.V.	
			2	8												
			5	7												MC
Saederup, 2008[86]	*Ccr2*$^{-/-}$	*Ccr2*$^{+/+}$	8	7	T/TAA	C57BL/6, *Apoe*$^{-/-}$	F	6	WD (Harlan Teklad TD-88137, 21% fat, 0.15%,	8	NA	NA	NA	NA	NA	Lesion size
			11	10		C57BL/6, *Apoe*$^{-/-}$, *Cx3cl1*$^{-/-}$										
			8	9	Aortic root	C57BL/6, *Apoe*$^{-/-}$										

First author, year	Experimental groups	Control group(s)	Number of animals (Intervention)	Number of animals (control)	Lesion site	Genotype	Sex	Age at start of diet (wks)	Type of diet used	Duration of diet (wks)	Start of treatment (wks)	Dose	Duration of administration (wks)	Frequency of administration	Route of administration	Primary outcome(s)
			11	10		C57BL/6, $Apoe^{-/-}$, $Cx3cl1^{-/-}$			0%cholate)							
			8	9		C57BL/6, $Apoe^{-/-}$										MC
			11	10		C57BL/6, $Apoe^{-/-}$, $Cx3cl1^{-/-}$										
			9	11	T/TAA	C57BL/6, $Apoe^{-/-}$	M									Lesion size
			8	10		C57BL/6, $Apoe^{-/-}$, $Cx3cl1^{-/-}$										
			5	5	Aortic root	C57BL/6, $Apoe^{-/-}$										
			4	4		C57BL/6, $Apoe^{-/-}$, $Cx3cl1^{-/-}$										
Schober, 2002[79]	Met-RANTES	PBS vehicle	6	6	Carotid artery	C57BL/6, $Apoe^{-/-}$	NS	NA	WD (21% fat)	5	NA	10 ug	4	Daily	I.P.	Lesion size
			6	6												MC
Schober, 2004[80]	$Ccr2^{-/-}$	$Ccr2^{+/+}$	5	5	Carotid artery	C57BL/6-129/Ola, $Apoe^{-/-}$	F	8	WD (Altromin, 21% fat, 0.15% cholesterol, 19.5% casein)	5	NA	NA	NA	NA	NA	Lesion size
			5	5												MC
			5	5												SMC content
			5	5												Collagen content
Soehnlein, 2013[42]	$Ccr1^{-/-}$	$Ccr1^{+/+}$	5	5	Aortic root	C57BL/6, $Apoe^{-/-}$	M	6	WD (Altromin, 21% fat, 0.15% cholesterol)	4	NA	NA	NA	NA	NA	Lesion size
	$Ccr2^{-/-}$	$Ccr2^{+/+}$	5	5												
	$Ccr5^{-/-}$	$Ccr5^{+/+}$	5	5												
	$Ccr1^{-/-}$	$Ccr1^{+/+}$	13	13						8						
	$Ccr2^{-/-}$	$Ccr2^{+/+}$	13	13												
	$Ccr5^{-/-}$	$Ccr5^{+/+}$	13	13												
van Wanrooij, 2005[72]	TAK-779(CCR5/CXCR3 antagonist)	Vehicle (5% mannitol)	8	8	Aortic root	NA, $Ldlr^{-/-}$	F	15	WD (15% cocoa butter, 0.25% cholesterol)	6	17	150 ug/100µl	6	e/o/d	S.C.	Lesion size
			8	8						6						MC
			10	10	Carotid artery					8						Lesion size
			10	10						8						MC

First author, year	Experimental groups	Control group (s)	Number of animals (Intervention)	Number of animals (control)	Lesion site	Genotype	Sex	Age at start of diet (wks)	Type of diet used	Duration of diet (wks)	Start of treatment (wks)	Dose	Duration of administration (wks)	Frequency of administration	Route of administration	Primary outcome(s)
			10	10						8						Collagen content
			10	10						8						SMC content
Veillard, 2004[43]	Met-RANTES - (CCR1/CCR5 antagonist)	Control (without treatment)	8	8	Aortic root	C57BL/6J, *Ldlr*$^{-/-}$	M	12	WD (Research Diets D-12108,1.25% cholesterol, 0% cholate)	14	12	100 µg	14	2x/week	I.P.	Lesion size
		Saline (PBS)	8	8												
		Control (without treatment)	8	8	T/TAA											
		Saline (PBS)	8	8												
		Control (without treatment)	8	8	Aortic root											MC
			8	8												SMC content
			8	8												Collagen content
Veillard, 2005[87]	*Ccr2*$^{-/-}$	*Ccr2*$^{+/+}$	8	8	Aortic root	C57BL/6J, *Apoe*$^{-/-}$	M	8	WD (Research Diets D-12108,1.25% cholesterol, 0% cholate)	10	NA	NA	NA	NA	NA	Lesion size
			8	8	T/TAA											
			4	4	Aortic root											MC
			4	4												SMC content
			8	8		C57BL/6J, CXCR3$^{-/-}$, *Apoe*$^{-/-}$										Lesion size
			8	8	T/TAA											
			4	4	Aortic root											MC
			4	4												SMC content
Winter, 2018[91]	RS102895 (CCR2 antagonist)	Vehicle control	8	8	Aortic root	C57BL/6, *Apoe*$^{-/-}$	M/F	8	WD (Ssniff E15721-347, 21% fat and 0.15% cholesterol)	4	8	5 mg/kg	4	1x Daily	I.P.	Lesion size
			8	8												MC
			8	8												Lesion size
			8	8												MC
Yamashita, 2002[51]	Propagermanium (CCR2	Control (non-treated)	8	8	Aortic root	C57BL/6, *Apoe*$^{-/-}$	M/F	4	WD (Oriental Yeast,	8	4	5 mg/kg	8	Daily	Oral (Admixe	Lesion size
			8	8												MC

First author, year	Experimental groups	Control group (s)	Number of animals (Intervention)	Number of animals (control)	Lesion site	Genotype	Sex	Age at start of diet (wks)	Type of diet used	Duration of diet (wks)	Start of treatment (wks)	Dose	Duration of administration (wks)	Frequency of administration	Route of administration	Primary outcome(s)
	antagonist)		8	8					7.5% cocoa butter, 7.5%casein,1.25 % cholesterol, 0.5% sodium cholate)						d with diet)	
			9	8	T/TAA											
			8	8	Aortic root					12			12			Lesion size
Zernecke, 2006[50]	*Ccr5*$^{-/-}$	*Ccr5*$^{+/+}$	5	5	Aortic root	C57BL/6, *Apoe*$^{-/-}$		8-11	WD	12	NA	NA	NA	NA	NA	Lesion size
	Ccr5$^{-/-}$	*Ccr5*$^{+/+}$	5	6	Carotid artery					5						
	Ccr5$^{-/-}$	*Ccr5*$^{+/+}$	4	4												
	Ccr1$^{-/-}$	*Ccr1*$^{+/+}$	4	5												
	Ccr1$^{-/-}$	*Ccr1*$^{+/+}$	4	4												
	Ccr5$^{-/-}$	*Ccr5*$^{+/+}$	5	5												MC
	Ccr5$^{-/-}$	*Ccr5*$^{+/+}$	4	4												
	Ccr1$^{-/-}$	*Ccr1*$^{+/+}$	5	5												
	Ccr1$^{-/-}$	*Ccr1*$^{+/+}$	4	4												

BM, bone marrow; e/o/d, every other day; CCR1, CC-chemokine receptor 1; CCR2, CC-chemokine receptor 2; CCR5, CC-chemokine receptor 5; CD, Chow Diet; F, Female; I.M., intramuscular injection; I.P., intraperitoneal injection; I.R., Ischaemia Reperfusion; I.V., Intravenous injection; M, Male; MC, Macrophage content; NA, information not available; P.O., oral gavage; S.C, subcutaneous injection; T/TAA, Thoracic/Thoraco-abdominal aorta; WD, Western-type Diet.

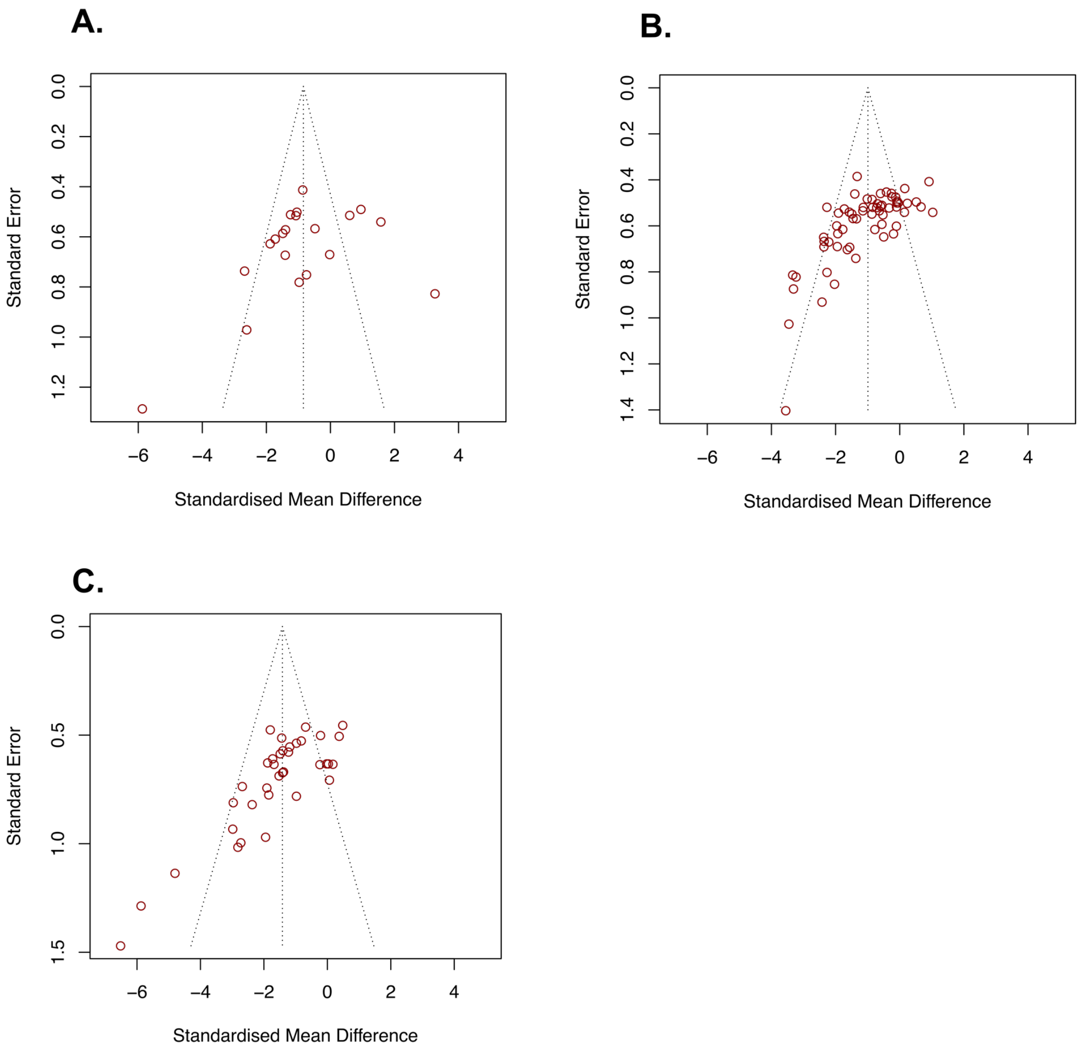

Figure S1. Funnel plots of meta-analysed studies with pseudo 95% confidence intervals assessing atherosclerotic lesion size. Red circles represent studies targeting (A) chemokine receptor 1, (B) chemokine receptor 2, (C) chemokine receptor 5.

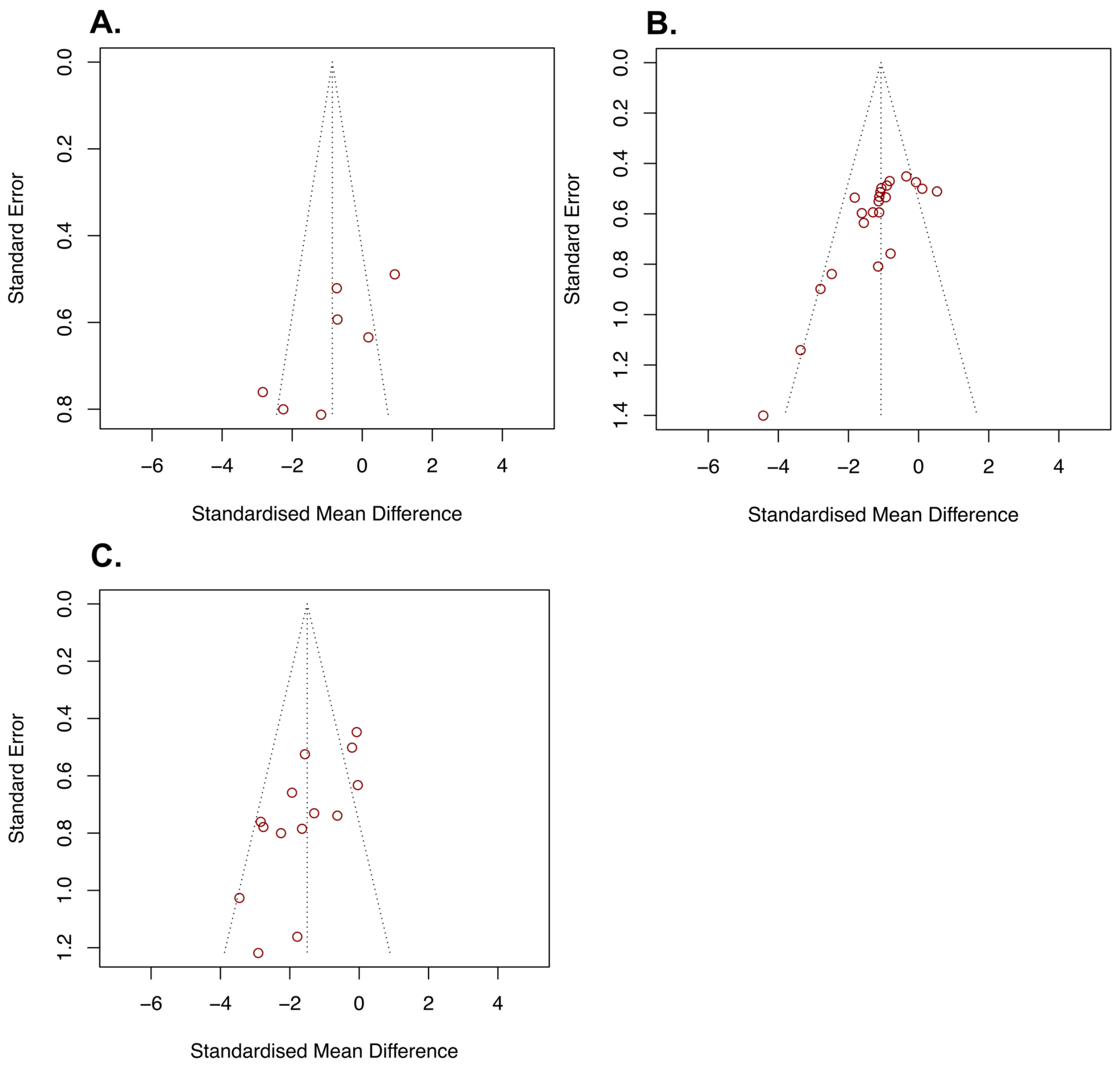

Figure S2. Funnel plots of meta-analysed studies with pseudo 95% confidence intervals assessing macrophage content in atherosclerotic plaque. Red circles represent studies targeting (A) chemokine receptor 1, (B) chemokine receptor 2, (C) chemokine receptor 5.

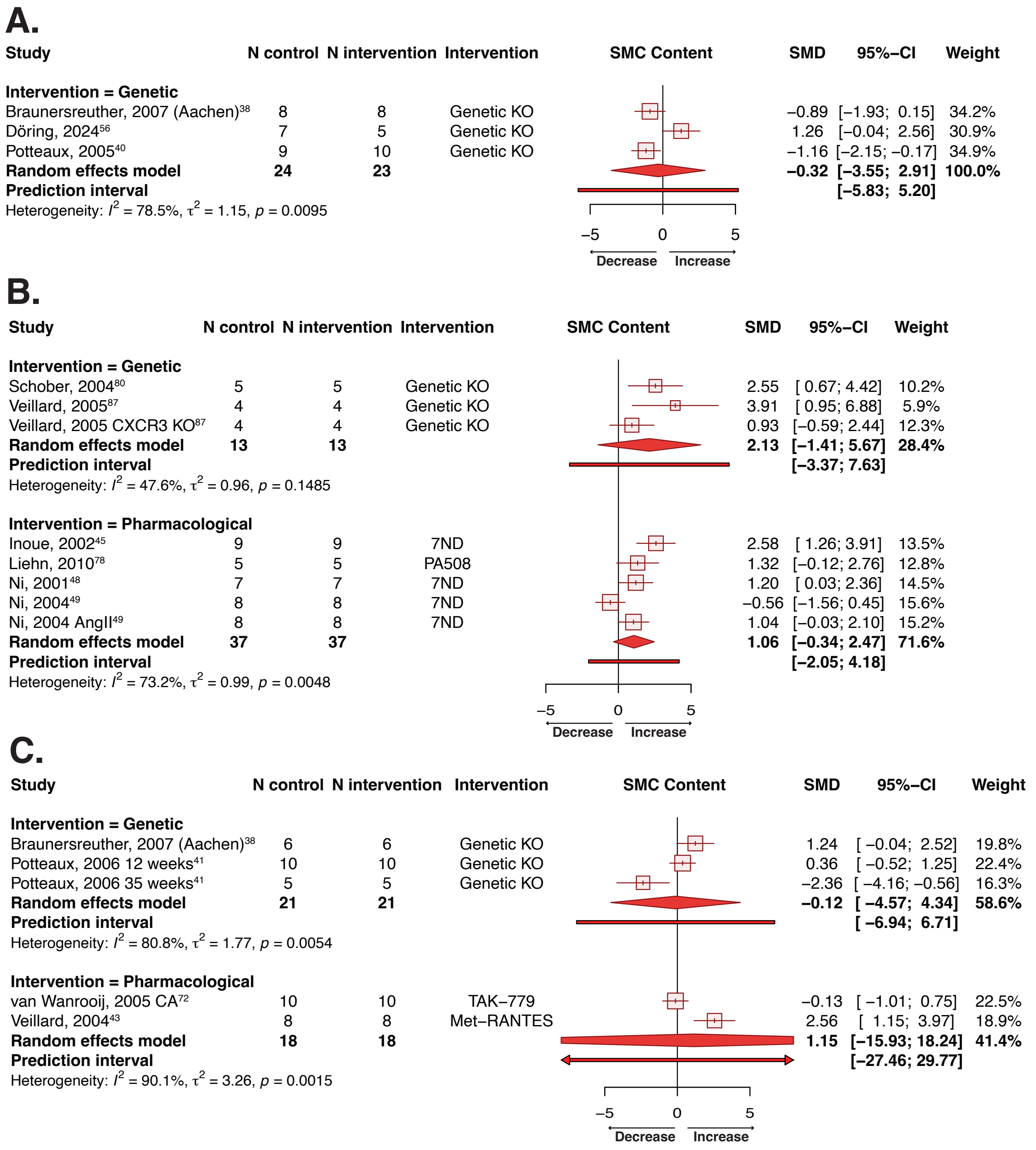

Figure S3. Forest plots showing the pooled estimate as a standardised mean difference (SMD) with corresponding 95% confidence intervals (CI) and 95% prediction intervals for the effects of targeting (A) chemokine receptor 1, (B) chemokine receptor 2 and (C) chemokine receptor 5 on smooth muscle cell (SMC) content in atherosclerotic plaque. I^2 and τ^2 heterogeneity statistics are provided. (n) weeks indicates the duration on diet. AngII, angiotensin II; CA, carotid artery; CXCR3, C-X-C motif chemokine receptor 3.

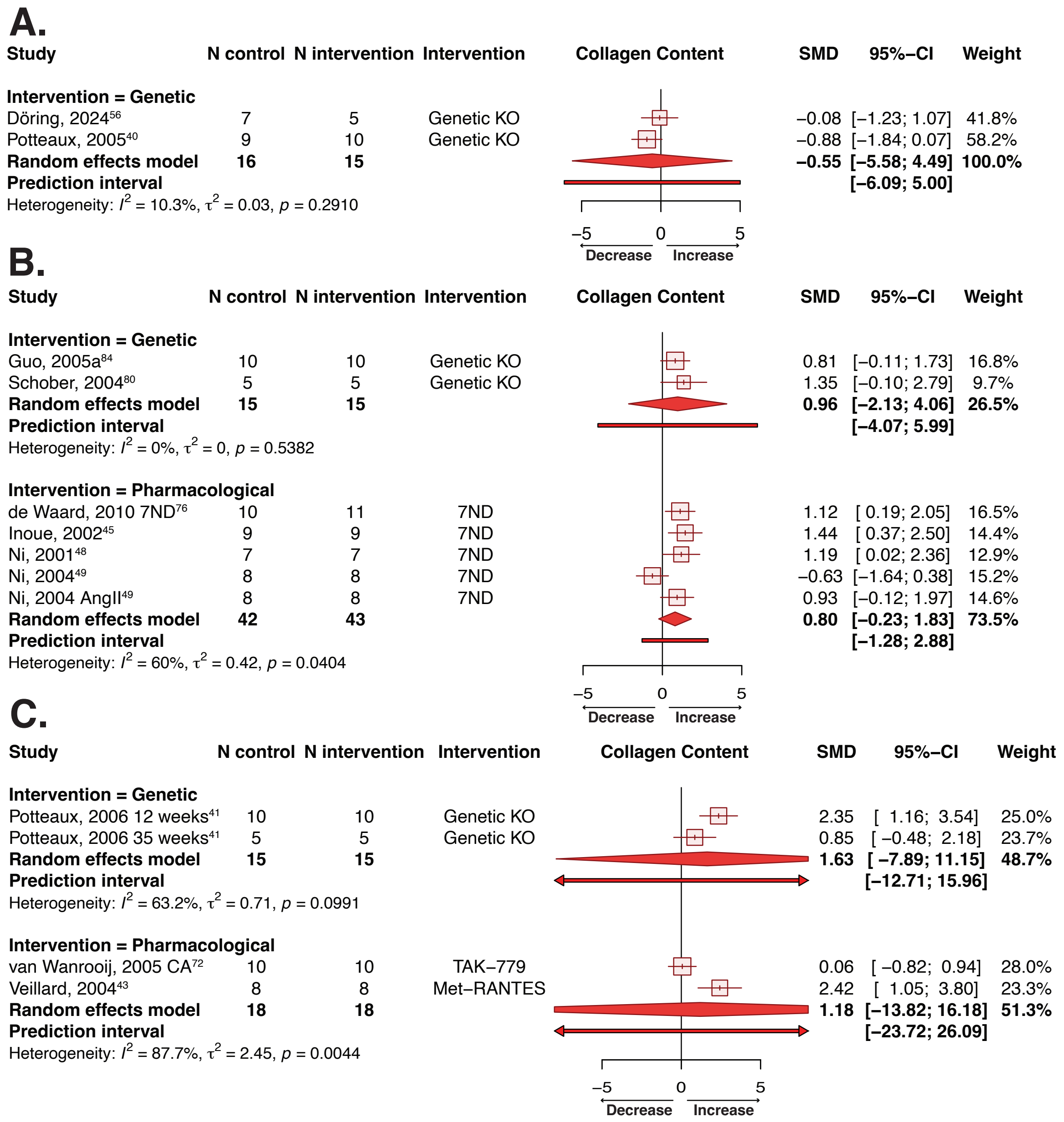

Figure S4. Forest plots showing the pooled estimate as a standardised mean difference (SMD) with corresponding 95% confidence intervals (CI) and 95% prediction intervals for the effects of targeting (A) chemokine receptor 1, (B) chemokine receptor 2 and (C) chemokine receptor 5 on collagen content in atherosclerotic plaque. I^2 and τ^2 heterogeneity statistics are provided. (n) weeks indicates the duration on diet. AngII, angiotensin II; CA, carotid artery.

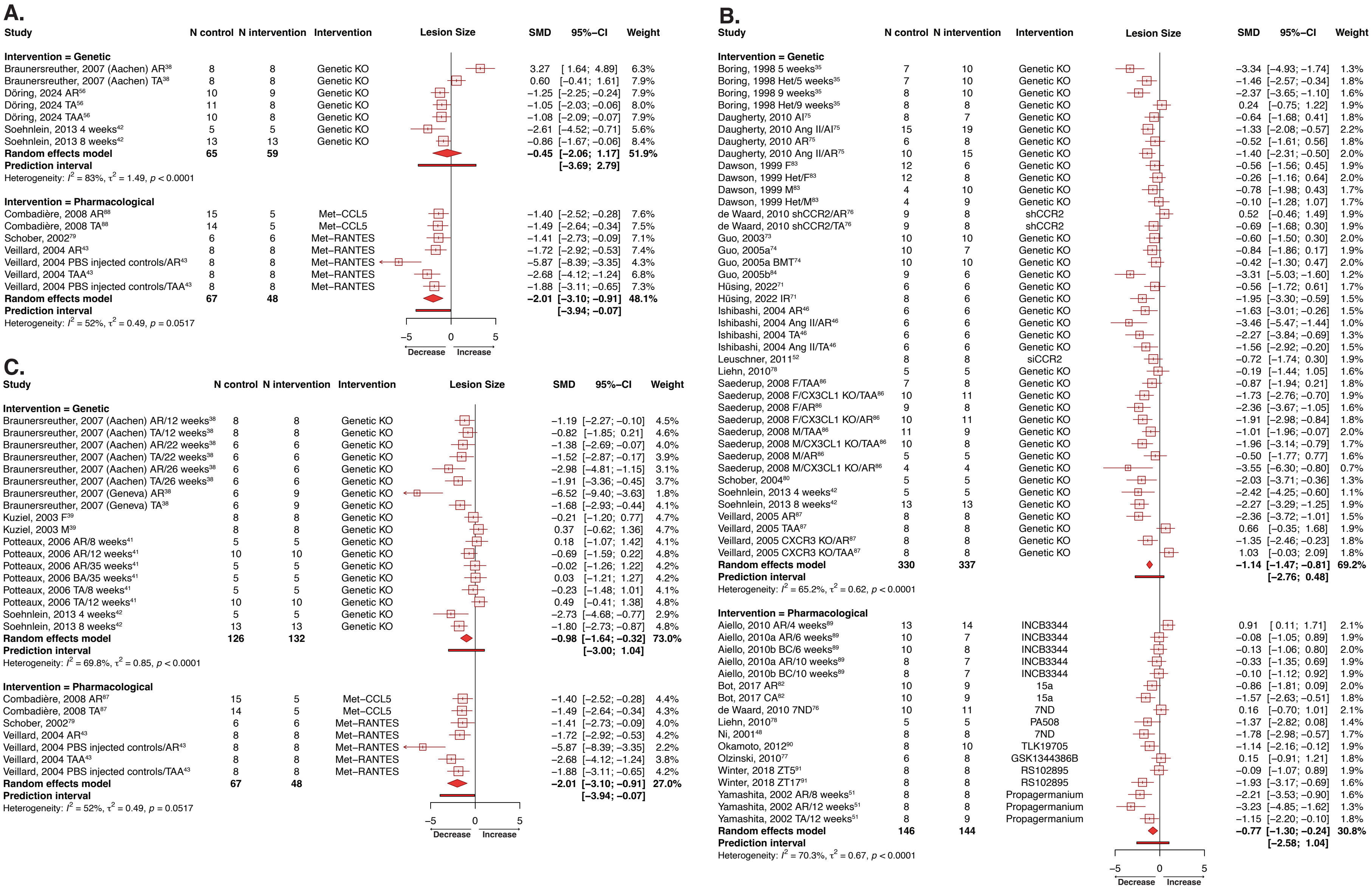
A.
Study | N control | N intervention | Intervention | Lesion Size | SMD | 95%–CI | Weight
Intervention = Genetic
Braunersreuther, 2007 (Aachen) AR[38] | 8 | 8 | Genetic KO | 3.27 | [1.64; 4.89] | 6.3%
Braunersreuther, 2007 (Aachen) TA[38] | 8 | 8 | Genetic KO | 0.60 | [−0.41; 1.61] | 7.9%
Döring, 2024 AR[56] | 10 | 9 | Genetic KO | −1.25 | [−2.25; −0.24] | 7.9%
Döring, 2024 TA[56] | 11 | 8 | Genetic KO | −1.05 | [−2.03; −0.06] | 8.0%
Döring, 2024 TAA[56] | 10 | 8 | Genetic KO | −1.08 | [−2.09; −0.07] | 7.9%
Soehnlein, 2013 4 weeks[42] | 5 | 5 | Genetic KO | −2.61 | [−4.52; −0.71] | 5.6%
Soehnlein, 2013 8 weeks[42] | 13 | 13 | Genetic KO | −0.86 | [−1.67; −0.06] | 8.4%
Random effects model | 65 | 59 | −0.45 | [−2.06; 1.17] | 51.9%
Prediction interval | [−3.69; 2.79]
Heterogeneity: I^2 = 83%, τ^2 = 1.49, p < 0.0001
Intervention = Pharmacological
Combadière, 2008 AR[88] | 15 | 5 | Met–CCL5 | −1.40 | [−2.52; −0.28] | 7.6%
Combadière, 2008 TA[88] | 14 | 5 | Met–CCL5 | −1.49 | [−2.64; −0.34] | 7.5%
Schober, 2002[79] | 6 | 6 | Met–RANTES | −1.41 | [−2.73; −0.09] | 7.1%
Veillard, 2004 AR[43] | 8 | 8 | Met–RANTES | −1.72 | [−2.92; −0.53] | 7.4%
Veillard, 2004 PBS injected controls/AR[43] | 8 | 8 | Met–RANTES | −5.87 | [−8.39; −3.35] | 4.3%
Veillard, 2004 TAA[43] | 8 | 8 | Met–RANTES | −2.68 | [−4.12; −1.24] | 6.8%
Veillard, 2004 PBS injected controls/TAA[43] | 8 | 8 | Met–RANTES | −1.88 | [−3.11; −0.65] | 7.3%
Random effects model | 67 | 48 | −2.01 | [−3.10; −0.91] | 48.1%
Prediction interval | [−3.94; −0.07]
Heterogeneity: I^2 = 52%, τ^2 = 0.49, p = 0.0517
−5 0 5
Decrease Increase
B.
Study | N control | N intervention | Intervention | Lesion Size | SMD | 95%–CI | Weight
Intervention = Genetic
Boring, 1998 5 weeks[35] | 7 | 10 | Genetic KO | −3.34 | [−4.93; −1.74] | 1.3%
Boring, 1998 Het/5 weeks[35] | 7 | 10 | Genetic KO | −1.46 | [−2.57; −0.34] | 1.8%
Boring, 1998 9 weeks[35] | 8 | 10 | Genetic KO | −2.37 | [−3.65; −1.10] | 1.6%
Boring, 1998 Het/9 weeks[35] | 8 | 8 | Genetic KO | 0.24 | [−0.75; 1.22] | 1.9%
Daugherty, 2010 AI[75] | 8 | 7 | Genetic KO | −0.64 | [−1.68; 0.41] | 1.8%
Daugherty, 2010 Ang II/AI[75] | 15 | 19 | Genetic KO | −1.33 | [−2.08; −0.57] | 2.2%
Daugherty, 2010 AR[75] | 6 | 8 | Genetic KO | −0.52 | [−1.61; 0.56] | 1.8%
Daugherty, 2010 Ang II/AR[75] | 10 | 15 | Genetic KO | −1.40 | [−2.31; −0.50] | 2.0%
Dawson, 1999 F[83] | 12 | 6 | Genetic KO | −0.56 | [−1.56; 0.45] | 1.9%
Dawson, 1999 Het/F[83] | 12 | 8 | Genetic KO | −0.26 | [−1.16; 0.64] | 2.0%
Dawson, 1999 M[83] | 4 | 10 | Genetic KO | −0.78 | [−1.98; 0.43] | 1.7%
Dawson, 1999 Het/M[83] | 4 | 9 | Genetic KO | −0.10 | [−1.28; 1.07] | 1.7%
de Waard, 2010 shCCR2/AR[76] | 9 | 8 | shCCR2 | 0.52 | [−0.46; 1.49] | 1.9%
de Waard, 2010 shCCR2/TA[76] | 9 | 8 | shCCR2 | −0.69 | [−1.68; 0.30] | 1.9%
Guo, 2003[73] | 10 | 10 | Genetic KO | −0.60 | [−1.50; 0.30] | 2.0%
Guo, 2005a[74] | 10 | 7 | Genetic KO | −0.84 | [−1.86; 0.17] | 1.9%
Guo, 2005a BMT[74] | 10 | 10 | Genetic KO | −0.42 | [−1.30; 0.47] | 2.0%
Guo, 2005b[84] | 9 | 6 | Genetic KO | −3.31 | [−5.03; −1.60] | 1.2%
Hüsing, 2022[71] | 6 | 6 | Genetic KO | −0.56 | [−1.72; 0.61] | 1.7%
Hüsing, 2022 IR[71] | 8 | 6 | Genetic KO | −1.95 | [−3.30; −0.59] | 1.5%
Ishibashi, 2004 AR[46] | 6 | 6 | Genetic KO | −1.63 | [−3.01; −0.26] | 1.5%
Ishibashi, 2004 Ang II/AR[46] | 6 | 6 | Genetic KO | −3.46 | [−5.47; −1.44] | 1.0%
Ishibashi, 2004 TA[46] | 6 | 6 | Genetic KO | −2.27 | [−3.84; −0.69] | 1.3%
Ishibashi, 2004 Ang II/TA[46] | 6 | 6 | Genetic KO | −1.56 | [−2.92; −0.20] | 1.5%
Leuschner, 2011[52] | 8 | 8 | siCCR2 | −0.72 | [−1.74; 0.30] | 1.9%
Liehn, 2010[78] | 5 | 5 | Genetic KO | −0.19 | [−1.44; 1.05] | 1.6%
Saederup, 2008 F/TAA[86] | 7 | 8 | Genetic KO | −0.87 | [−1.94; 0.21] | 1.8%
Saederup, 2008 F/CX3CL1 KO/TAA[86] | 10 | 11 | Genetic KO | −1.73 | [−2.76; −0.70] | 1.9%
Saederup, 2008 F/AR[86] | 9 | 8 | Genetic KO | −2.36 | [−3.67; −1.05] | 1.6%
Saederup, 2008 F/CX3CL1 KO/AR[86] | 10 | 11 | Genetic KO | −1.91 | [−2.98; −0.84] | 1.8%
Saederup, 2008 M/TAA[86] | 11 | 9 | Genetic KO | −1.01 | [−1.96; −0.07] | 2.0%
Saederup, 2008 M/CX3CL1 KO/TAA[86] | 10 | 8 | Genetic KO | −1.96 | [−3.14; −0.79] | 1.7%
Saederup, 2008 M/AR[86] | 5 | 5 | Genetic KO | −0.50 | [−1.77; 0.77] | 1.6%
Saederup, 2008 M/CX3CL1 KO/AR[86] | 4 | 4 | Genetic KO | −3.55 | [−6.30; −0.80] | 0.7%
Schober, 2004[80] | 5 | 5 | Genetic KO | −2.03 | [−3.71; −0.36] | 1.3%
Soehnlein, 2013 4 weeks[42] | 5 | 5 | Genetic KO | −2.42 | [−4.25; −0.60] | 1.1%
Soehnlein, 2013 8 weeks[42] | 13 | 13 | Genetic KO | −2.27 | [−3.29; −1.25] | 1.9%
Veillard, 2005 AR[87] | 8 | 8 | Genetic KO | −2.36 | [−3.72; −1.01] | 1.5%
Veillard, 2005 TAA[87] | 8 | 8 | Genetic KO | 0.66 | [−0.35; 1.68] | 1.9%
Veillard, 2005 CXCR3 KO/AR[87] | 8 | 8 | Genetic KO | −1.35 | [−2.46; −0.23] | 1.8%
Veillard, 2005 CXCR3 KO/TAA[87] | 8 | 8 | Genetic KO | 1.03 | [−0.03; 2.09] | 1.8%
Random effects model | 330 | 337 | −1.14 | [−1.47; −0.81] | 69.2%
Prediction interval | [−2.76; 0.48]
Heterogeneity: I^2 = 65.2%, τ^2 = 0.62, p < 0.0001
Intervention = Pharmacological
Aiello, 2010 AR/4 weeks[89] | 13 | 14 | INCB3344 | 0.91 | [0.11; 1.71] | 2.1%
Aiello, 2010a AR/6 weeks[89] | 10 | 7 | INCB3344 | −0.08 | [−1.05; 0.89] | 1.9%
Aiello, 2010b BC/6 weeks[89] | 10 | 8 | INCB3344 | −0.13 | [−1.06; 0.80] | 2.0%
Aiello, 2010a AR/10 weeks[89] | 8 | 7 | INCB3344 | −0.33 | [−1.35; 0.69] | 1.9%
Aiello, 2010b BC/10 weeks[89] | 8 | 7 | INCB3344 | −0.10 | [−1.12; 0.92] | 1.9%
Bot, 2017 AR[82] | 10 | 9 | 15a | −0.86 | [−1.81; 0.09] | 2.0%
Bot, 2017 CA[82] | 10 | 9 | 15a | −1.57 | [−2.63; −0.51] | 1.8%
de Waard, 2010 7ND[76] | 10 | 11 | 7ND | 0.16 | [−0.70; 1.01] | 2.1%
Liehn, 2010[78] | 5 | 5 | PA508 | −1.37 | [−2.82; 0.08] | 1.4%
Ni, 2001[48] | 8 | 8 | 7ND | −1.78 | [−2.98; −0.57] | 1.7%
Okamoto, 2012[90] | 8 | 10 | TLK19705 | −1.14 | [−2.16; −0.12] | 1.9%
Olzinski, 2010[77] | 6 | 8 | GSK1344386B | 0.15 | [−0.91; 1.21] | 1.8%
Winter, 2018 ZT5[91] | 8 | 8 | RS102895 | −0.09 | [−1.07; 0.89] | 1.9%
Winter, 2018 ZT17[91] | 8 | 8 | RS102895 | −1.93 | [−3.17; −0.69] | 1.6%
Yamashita, 2002 AR/8 weeks[51] | 8 | 8 | Propagermanium | −2.21 | [−3.53; −0.90] | 1.6%
Yamashita, 2002 AR/12 weeks[51] | 8 | 8 | Propagermanium | −3.23 | [−4.85; −1.62] | 1.3%
Yamashita, 2002 TA/12 weeks[51] | 8 | 9 | Propagermanium | −1.15 | [−2.20; −0.10] | 1.8%
Random effects model | 146 | 144 | −0.77 | [−1.30; −0.24] | 30.8%
Prediction interval | [−2.58; 1.04]
Heterogeneity: I^2 = 70.3%, τ^2 = 0.67, p < 0.0001
−5 0 5
Decrease Increase
C.
Study | N control | N intervention | Intervention | Lesion Size | SMD | 95%–CI | Weight
Intervention = Genetic
Braunersreuther, 2007 (Aachen) AR/12 weeks[38] | 8 | 8 | Genetic KO | −1.19 | [−2.27; −0.10] | 4.5%
Braunersreuther, 2007 (Aachen) TA/12 weeks[38] | 8 | 8 | Genetic KO | −0.82 | [−1.85; 0.21] | 4.6%
Braunersreuther, 2007 (Aachen) AR/22 weeks[38] | 6 | 6 | Genetic KO | −1.38 | [−2.69; −0.07] | 4.0%
Braunersreuther, 2007 (Aachen) TA/22 weeks[38] | 6 | 6 | Genetic KO | −1.52 | [−2.87; −0.17] | 3.9%
Braunersreuther, 2007 (Aachen) AR/26 weeks[38] | 6 | 6 | Genetic KO | −2.98 | [−4.81; −1.15] | 3.1%
Braunersreuther, 2007 (Aachen) TA/26 weeks[38] | 6 | 6 | Genetic KO | −1.91 | [−3.36; −0.45] | 3.7%
Braunersreuther, 2007 (Geneva) AR[38] | 6 | 9 | Genetic KO | −6.52 | [−9.40; −3.63] | 1.8%
Braunersreuther, 2007 (Geneva) TA[38] | 6 | 9 | Genetic KO | −1.68 | [−2.93; −0.44] | 4.1%
Kuziel, 2003 F[39] | 8 | 8 | Genetic KO | −0.21 | [−1.20; 0.77] | 4.7%
Kuziel, 2003 M[39] | 8 | 8 | Genetic KO | 0.37 | [−0.62; 1.36] | 4.7%
Potteaux, 2006 AR/8 weeks[41] | 5 | 5 | Genetic KO | 0.18 | [−1.07; 1.42] | 4.1%
Potteaux, 2006 AR/12 weeks[41] | 10 | 10 | Genetic KO | −0.69 | [−1.59; 0.22] | 4.8%
Potteaux, 2006 AR/35 weeks[41] | 5 | 5 | Genetic KO | −0.02 | [−1.26; 1.22] | 4.2%
Potteaux, 2006 BA/35 weeks[41] | 5 | 5 | Genetic KO | 0.03 | [−1.21; 1.27] | 4.2%
Potteaux, 2006 TA/8 weeks[41] | 5 | 5 | Genetic KO | −0.23 | [−1.48; 1.01] | 4.1%
Potteaux, 2006 TA/12 weeks[41] | 10 | 10 | Genetic KO | 0.49 | [−0.41; 1.38] | 4.8%
Soehnlein, 2013 4 weeks[42] | 5 | 5 | Genetic KO | −2.73 | [−4.68; −0.77] | 2.9%
Soehnlein, 2013 8 weeks[42] | 13 | 13 | Genetic KO | −1.80 | [−2.73; −0.87] | 4.8%
Random effects model | 126 | 132 | −0.98 | [−1.64; −0.32] | 73.0%
Prediction interval | [−3.00; 1.04]
Heterogeneity: I^2 = 69.8%, τ^2 = 0.85, p < 0.0001
Intervention = Pharmacological
Combadière, 2008 AR[87] | 15 | 5 | Met–CCL5 | −1.40 | [−2.52; −0.28] | 4.4%
Combadière, 2008 TA[87] | 14 | 5 | Met–CCL5 | −1.49 | [−2.64; −0.34] | 4.3%
Schober, 2002[79] | 6 | 6 | Met–RANTES | −1.41 | [−2.73; −0.09] | 4.0%
Veillard, 2004 AR[43] | 8 | 8 | Met–RANTES | −1.72 | [−2.92; −0.53] | 4.2%
Veillard, 2004 PBS injected controls/AR[43] | 8 | 8 | Met–RANTES | −5.87 | [−8.39; −3.35] | 2.2%
Veillard, 2004 TAA[43] | 8 | 8 | Met–RANTES | −2.68 | [−4.12; −1.24] | 3.8%
Veillard, 2004 PBS injected controls/TAA[43] | 8 | 8 | Met–RANTES | −1.88 | [−3.11; −0.65] | 4.2%
Random effects model | 67 | 48 | −2.01 | [−3.10; −0.91] | 27.0%
Prediction interval | [−3.94; −0.07]
Heterogeneity: I^2 = 52%, τ^2 = 0.49, p = 0.0517
−5 0 5
Decrease Increase

Figure S5. Sensitivity analyses conducted excluding high risk of bias studies. Forest plots show the pooled estimate as a standardised mean difference (SMD) and corresponding 95% confidence intervals (CI) for the effects of targeting (A) chemokine receptor 1, (B) chemokine receptor 2 and (C) chemokine receptor 5 on lesion size. I^2 and τ^2 heterogeneity statistics are provided. (n) weeks indicates the duration on diet. AI, aortic intima; AngII, angiotensin II; AR, aortic root; BC, brachiocephalic artery; BMT, bone marrow transplantation; CA, carotid artery; CXCR3, C-X-C motif chemokine receptor 3; CX3CL1, chemokine C-X3-C motif ligand 1; F, female; Het, heterozygous; IFNG mAb, interferon gamma monoclonal antibody; IL-10 mAb; interleukin-10 monoclonal antibody; IR, ischaemia reperfusion; KO, knock-out; M, male; MT4, metallothionein 4; TA, thoracic aorta; TAA, thoracic/abdominal aorta; PBS, phosphate-buffered saline.

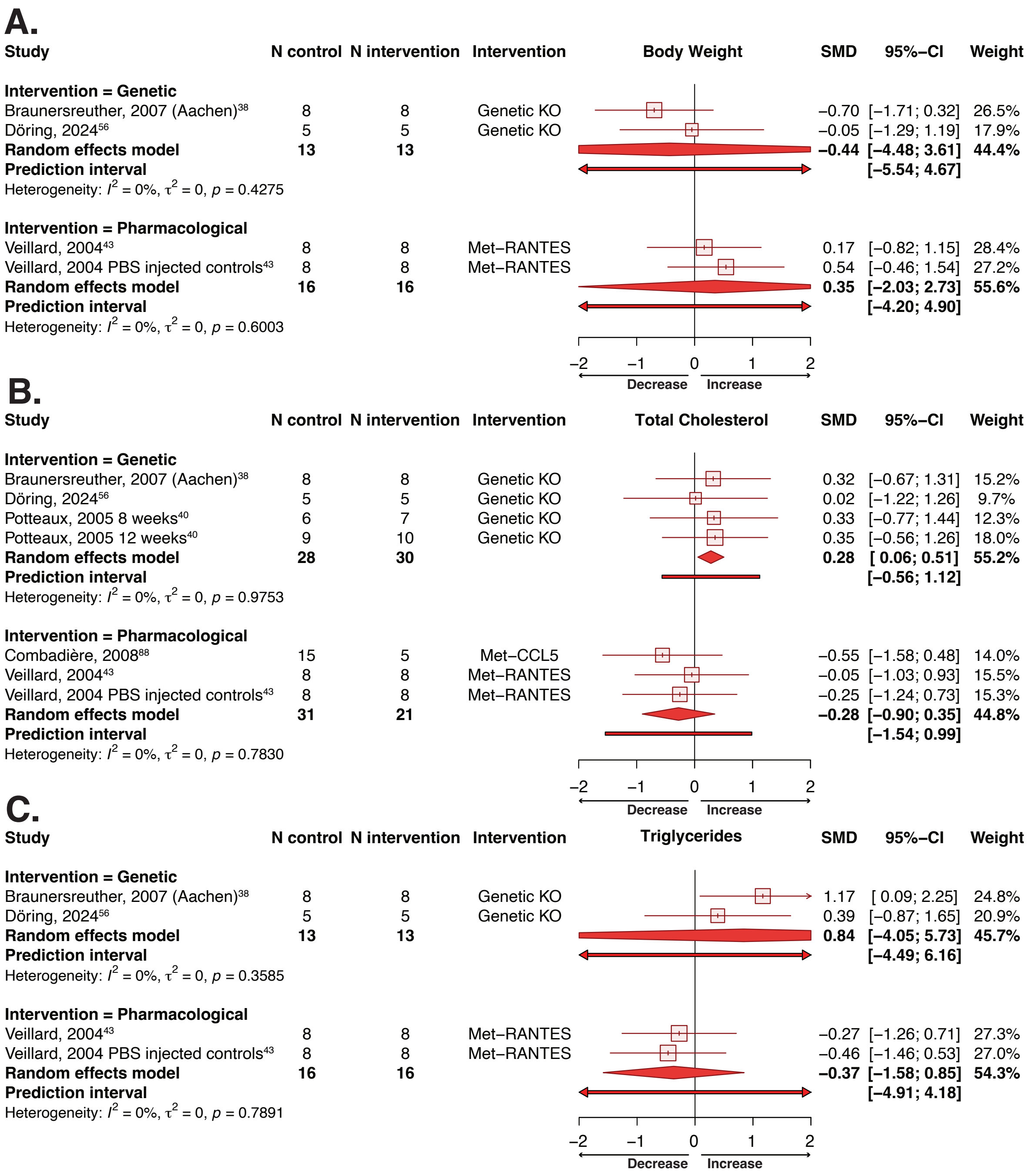

Figure S6: Forest plots showing the pooled estimate as a standardised mean difference (SMD) and corresponding 95% confidence intervals (CI) for the effect of targeting chemokine receptor 1 on (A) animal body weight, (B) total cholesterol, and (C) triglycerides. I^2 and τ^2 heterogeneity statistics are provided. (n) weeks indicates the duration on diet. PBS, phosphate-buffered saline.

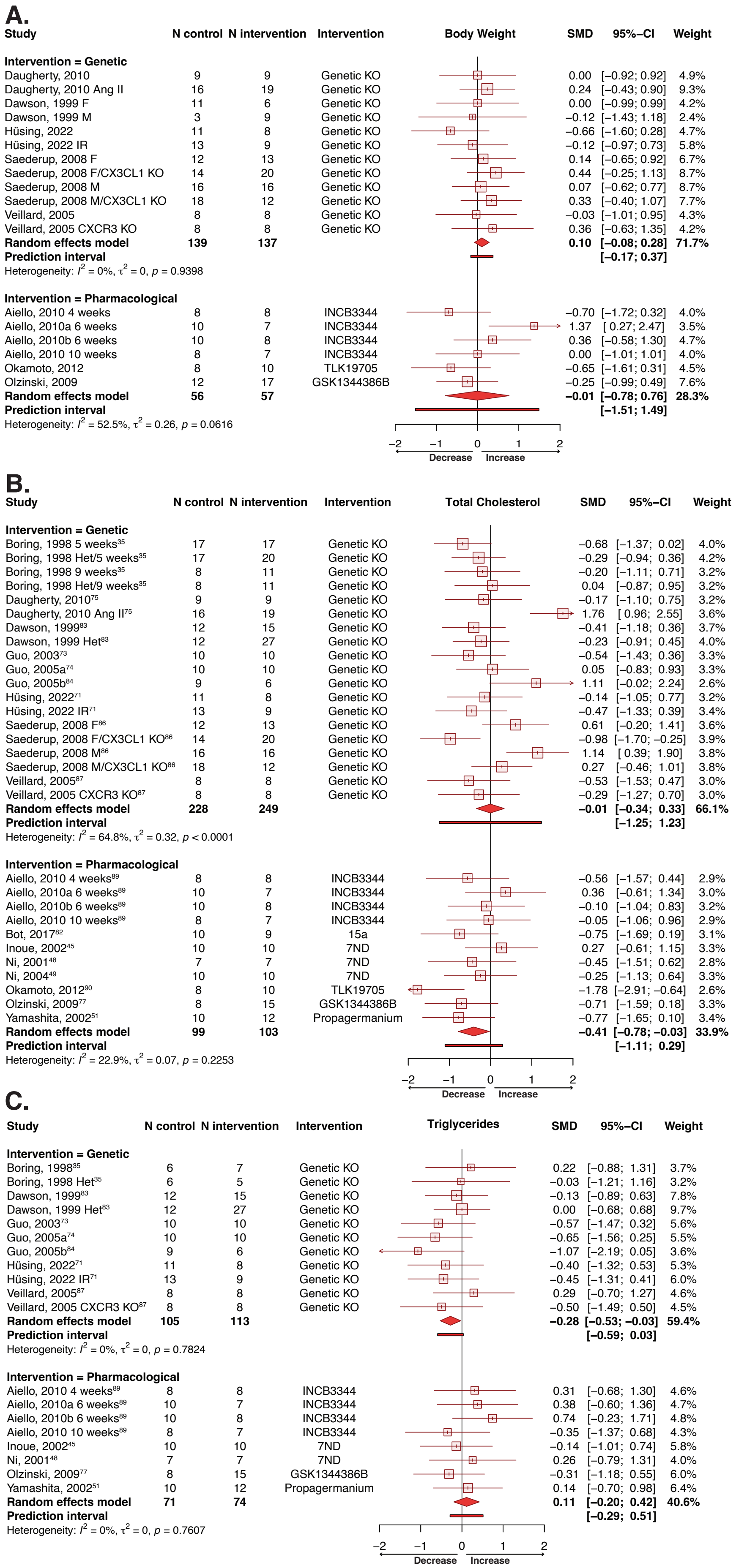
A.
Study N control N intervention Intervention Body Weight SMD 95%−CI Weight
Intervention = Genetic
Daugherty, 2010 9 9 Genetic KO 0.00 [−0.92; 0.92] 4.9%
Daugherty, 2010 Ang II 16 19 Genetic KO 0.24 [−0.43; 0.90] 9.3%
Dawson, 1999 F 11 6 Genetic KO 0.00 [−0.99; 0.99] 4.2%
Dawson, 1999 M 3 9 Genetic KO −0.12 [−1.43; 1.18] 2.4%
Hüsing, 2022 11 8 Genetic KO −0.66 [−1.60; 0.28] 4.7%
Hüsing, 2022 IR 13 9 Genetic KO −0.12 [−0.97; 0.73] 5.8%
Saederup, 2008 F 12 13 Genetic KO 0.14 [−0.65; 0.92] 6.7%
Saederup, 2008 F/CX3CL1 KO 14 20 Genetic KO 0.44 [−0.25; 1.13] 8.7%
Saederup, 2008 M 16 16 Genetic KO 0.07 [−0.62; 0.77] 8.7%
Saederup, 2008 M/CX3CL1 KO 18 12 Genetic KO 0.33 [−0.40; 1.07] 7.7%
Veillard, 2005 8 8 Genetic KO −0.03 [−1.01; 0.95] 4.3%
Veillard, 2005 CXCR3 KO 8 8 Genetic KO 0.36 [−0.63; 1.35] 4.2%
Random effects model 139 137 0.10 [−0.08; 0.28] 71.7%
Prediction interval [−0.17; 0.37]
Heterogeneity: I^2 = 0%, τ^2 = 0, p = 0.9398
Intervention = Pharmacological
Aiello, 2010 4 weeks 8 8 INCB3344 −0.70 [−1.72; 0.32] 4.0%
Aiello, 2010a 6 weeks 10 7 INCB3344 1.37 [0.27; 2.47] 3.5%
Aiello, 2010b 6 weeks 10 8 INCB3344 0.36 [−0.58; 1.30] 4.7%
Aiello, 2010 10 weeks 8 7 INCB3344 0.00 [−1.01; 1.01] 4.0%
Okamoto, 2012 8 10 TLK19705 −0.65 [−1.61; 0.31] 4.5%
Olzinski, 2009 12 17 GSK1344386B −0.25 [−0.99; 0.49] 7.6%
Random effects model 56 57 −0.01 [−0.78; 0.76] 28.3%
Prediction interval [−1.51; 1.49]
Heterogeneity: I^2 = 52.5%, τ^2 = 0.26, p = 0.0616
−2 −1 0 1 2
Decrease Increase
B.
Study N control N intervention Intervention Total Cholesterol SMD 95%−CI Weight
Intervention = Genetic
Boring, 1998 5 weeks[35] 17 17 Genetic KO −0.68 [−1.37; 0.02] 4.0%
Boring, 1998 Het/5 weeks[35] 17 20 Genetic KO −0.29 [−0.94; 0.36] 4.2%
Boring, 1998 9 weeks[35] 8 11 Genetic KO −0.20 [−1.11; 0.71] 3.2%
Boring, 1998 Het/9 weeks[35] 8 11 Genetic KO 0.04 [−0.87; 0.95] 3.2%
Daugherty, 2010[75] 9 9 Genetic KO −0.17 [−1.10; 0.75] 3.2%
Daugherty, 2010 Ang II[75] 16 19 Genetic KO 1.76 [0.96; 2.55] 3.6%
Dawson, 1999[83] 12 15 Genetic KO −0.41 [−1.18; 0.36] 3.7%
Dawson, 1999 Het[83] 12 27 Genetic KO −0.23 [−0.91; 0.45] 4.0%
Guo, 2003[73] 10 10 Genetic KO −0.54 [−1.43; 0.36] 3.3%
Guo, 2005a[74] 10 10 Genetic KO 0.05 [−0.83; 0.93] 3.3%
Guo, 2005b[84] 9 6 Genetic KO 1.11 [−0.02; 2.24] 2.6%
Hüsing, 2022[71] 11 8 Genetic KO −0.14 [−1.05; 0.77] 3.2%
Hüsing, 2022 IR[71] 13 9 Genetic KO −0.47 [−1.33; 0.39] 3.4%
Saederup, 2008 F[86] 12 13 Genetic KO 0.61 [−0.20; 1.41] 3.6%
Saederup, 2008 F/CX3CL1 KO[86] 14 20 Genetic KO −0.98 [−1.70; −0.25] 3.9%
Saederup, 2008 M[86] 16 16 Genetic KO 1.14 [0.39; 1.90] 3.8%
Saederup, 2008 M/CX3CL1 KO[86] 18 12 Genetic KO 0.27 [−0.46; 1.01] 3.8%
Veillard, 2005[87] 8 8 Genetic KO −0.53 [−1.53; 0.47] 3.0%
Veillard, 2005 CXCR3 KO[87] 8 8 Genetic KO −0.29 [−1.27; 0.70] 3.0%
Random effects model 228 249 −0.01 [−0.34; 0.33] 66.1%
Prediction interval [−1.25; 1.23]
Heterogeneity: I^2 = 64.8%, τ^2 = 0.32, p < 0.0001
Intervention = Pharmacological
Aiello, 2010 4 weeks[89] 8 8 INCB3344 −0.56 [−1.57; 0.44] 2.9%
Aiello, 2010a 6 weeks[89] 10 7 INCB3344 0.36 [−0.61; 1.34] 3.0%
Aiello, 2010b 6 weeks[89] 10 8 INCB3344 −0.10 [−1.04; 0.83] 3.2%
Aiello, 2010 10 weeks[89] 8 7 INCB3344 −0.05 [−1.06; 0.96] 2.9%
Bot, 2017[82] 10 9 15a −0.75 [−1.69; 0.19] 3.1%
Inoue, 2002[45] 10 10 7ND 0.27 [−0.61; 1.15] 3.3%
Ni, 2001[48] 7 7 7ND −0.45 [−1.51; 0.62] 2.8%
Ni, 2004[49] 10 10 7ND −0.25 [−1.13; 0.64] 3.3%
Okamoto, 2012[90] 8 10 TLK19705 −1.78 [−2.91; −0.64] 2.6%
Olzinski, 2009[77] 8 15 GSK1344386B −0.71 [−1.59; 0.18] 3.3%
Yamashita, 2002[51] 10 12 Propagermanium −0.77 [−1.65; 0.10] 3.4%
Random effects model 99 103 −0.41 [−0.78; −0.03] 33.9%
Prediction interval [−1.11; 0.29]
Heterogeneity: I^2 = 22.9%, τ^2 = 0.07, p = 0.2253
−2 −1 0 1 2
Decrease Increase
C.
Study N control N intervention Intervention Triglycerides SMD 95%−CI Weight
Intervention = Genetic
Boring, 1998[35] 6 7 Genetic KO 0.22 [−0.88; 1.31] 3.7%
Boring, 1998 Het[35] 6 5 Genetic KO −0.03 [−1.21; 1.16] 3.2%
Dawson, 1999[83] 12 15 Genetic KO −0.13 [−0.89; 0.63] 7.8%
Dawson, 1999 Het[83] 12 27 Genetic KO 0.00 [−0.68; 0.68] 9.7%
Guo, 2003[73] 10 10 Genetic KO −0.57 [−1.47; 0.32] 5.6%
Guo, 2005a[74] 10 10 Genetic KO −0.65 [−1.56; 0.25] 5.5%
Guo, 2005b[84] 9 6 Genetic KO −1.07 [−2.19; 0.05] 3.6%
Hüsing, 2022[71] 11 8 Genetic KO −0.40 [−1.32; 0.53] 5.3%
Hüsing, 2022 IR[71] 13 9 Genetic KO −0.45 [−1.31; 0.41] 6.0%
Veillard, 2005[87] 8 8 Genetic KO 0.29 [−0.70; 1.27] 4.6%
Veillard, 2005 CXCR3 KO[87] 8 8 Genetic KO −0.50 [−1.49; 0.50] 4.5%
Random effects model 105 113 −0.28 [−0.53; −0.03] 59.4%
Prediction interval [−0.59; 0.03]
Heterogeneity: I^2 = 0%, τ^2 = 0, p = 0.7824
Intervention = Pharmacological
Aiello, 2010 4 weeks[89] 8 8 INCB3344 0.31 [−0.68; 1.30] 4.6%
Aiello, 2010a 6 weeks[89] 10 7 INCB3344 0.38 [−0.60; 1.36] 4.7%
Aiello, 2010b 6 weeks[89] 10 8 INCB3344 0.74 [−0.23; 1.71] 4.8%
Aiello, 2010 10 weeks[89] 8 7 INCB3344 −0.35 [−1.37; 0.68] 4.3%
Inoue, 2002[45] 10 10 7ND −0.14 [−1.01; 0.74] 5.8%
Ni, 2001[48] 7 7 7ND 0.26 [−0.79; 1.31] 4.0%
Olzinski, 2009[77] 8 15 GSK1344386B −0.31 [−1.18; 0.55] 6.0%
Yamashita, 2002[51] 10 12 Propagermanium 0.14 [−0.70; 0.98] 6.4%
Random effects model 71 74 0.11 [−0.20; 0.42] 40.6%
Prediction interval [−0.29; 0.51]
Heterogeneity: I^2 = 0%, τ^2 = 0, p = 0.7607
−2 −1 0 1 2
Decrease Increase

Figure S7: Forest plots showing the pooled estimate as a standardised mean difference (SMD) and corresponding 95% confidence intervals (CI) for the effect of targeting chemokine receptor 2 on (A) animal body weight, (B) total cholesterol, and (C) triglycerides. I^2 and τ^2 heterogeneity statistics are provided. (n) weeks indicates the duration on diet. AngII, angiotensin II; CXCR3, C-X-C motif chemokine receptor 3; CX3CL1, chemokine C-X3-C motif ligand 1; F, female; Het, heterozygous; IR, ischaemia reperfusion; KO, knock-out; M, male.

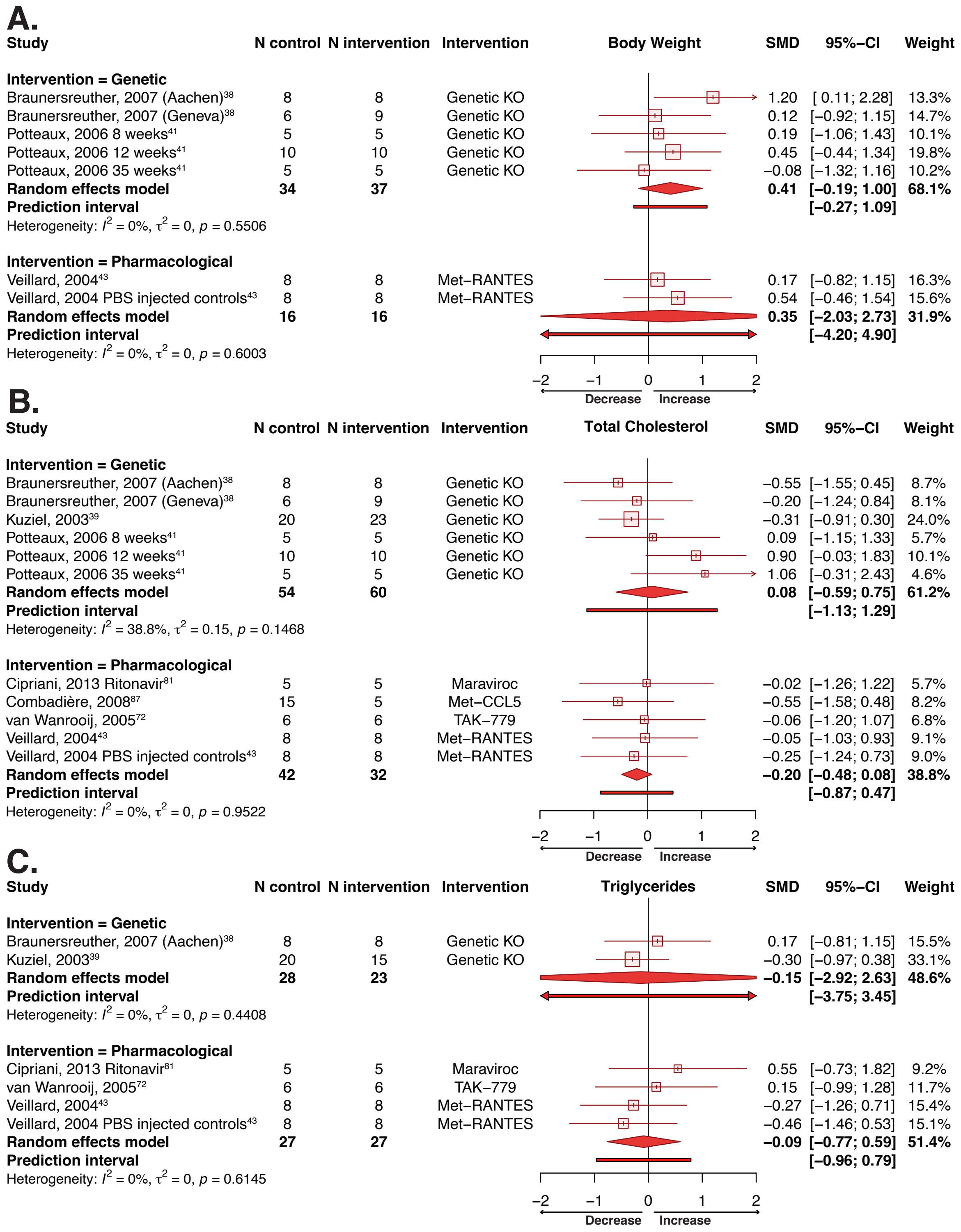

Figure S8: Forest plots showing the pooled estimate as a standardised mean difference (SMD) and corresponding 95% confidence intervals (CI) for the effect of targeting chemokine receptor 5 on (A) animal body weight, (B) total cholesterol, and (C) triglycerides. I^2 and τ^2 heterogeneity statistics are provided. (n) weeks indicates the duration on diet. PBS, phosphate-buffered saline.
